# Understanding the Degradation of a Model Si Anode in a Li-Ion Battery at the Atomic Scale

**DOI:** 10.1021/acs.jpclett.2c02236

**Published:** 2022-09-01

**Authors:** Se-Ho Kim, Kang Dong, Huan Zhao, Ayman A. El-Zoka, Xuyang Zhou, Eric V. Woods, Finn Giuliani, Ingo Manke, Dierk Raabe, Baptiste Gault

**Affiliations:** † Max-Planck Institut für Eisenforschung GmbH, Max-Planck-Straße 1, Düsseldorf 40237, Germany; ‡ Institute of Applied Materials, Helmholtz-Zentrum Berlin für Materialien und Energie, Berlin 14109, Germany; § Department of Materials, Royal School of Mines, Imperial College, London SW7 2AZ, United Kingdom

## Abstract

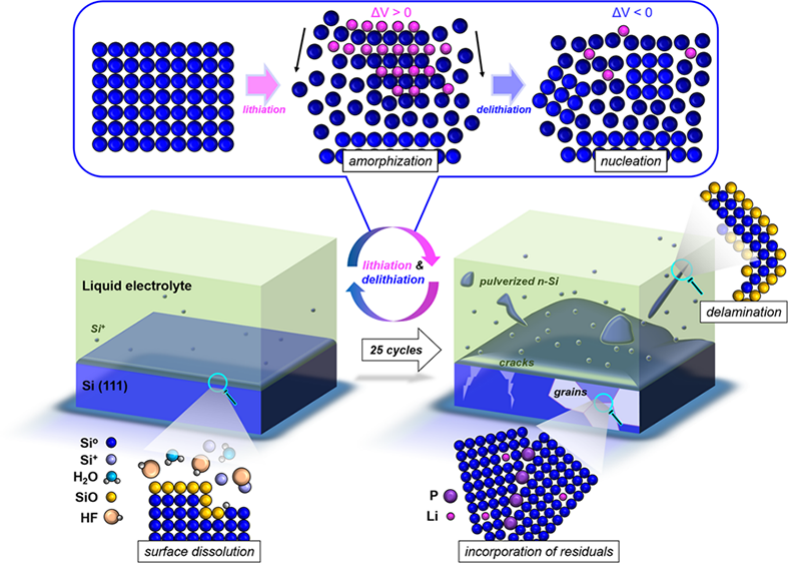

To advance the understanding of the degradation of the liquid electrolyte and Si electrode, and their interface, we exploit the latest developments in cryo-atom probe tomography. We evidence Si anode corrosion from the decomposition of the Li salt before charge–discharge cycles even begin. Volume shrinkage during delithiation leads to the development of nanograins from recrystallization in regions left amorphous by the lithiation. The newly created grain boundaries facilitate pulverization of nanoscale Si fragments, and one is found floating in the electrolyte. P is segregated to these grain boundaries, which confirms the decomposition of the electrolyte. As structural defects are bound to assist the nucleation of Li-rich phases in subsequent lithiations and accelerate the electrolyte’s decomposition, these insights into the developed nanoscale microstructure interacting with the electrolyte contribute to understanding the self-catalyzed/accelerated degradation Si anodes and can inform new battery designs unaffected by these life-limiting factors.

To meet the rapidly increasing demand for Li-ion batteries for electric vehicles,^1,2^ tremendous efforts have been devoted to discovering cheap and abundant anode materials that can replace graphite that is in short supply.^3^ A crystalline Si anode, which can offer nearly 10 times the capacity of a commercial graphite anode (*Q*_Si_ = 4200 mAh g^–1^ vs *Q*_graphite_ = 372 mAh g^–1^)^4^ has emerged as an attractive anode material for next-generation Li-ion batteries since the first development of the Li–Si anode by Lai in 1976.^5^ Compared to graphite, in which each of the six in-plane C atoms can only bond with one Li ion, each Si atom can bond with up to 4.4 Li ions.^6^ Thus, finding a path to exploiting Si as an anode material can be a revolutionary approach for reaching batteries with ultrahigh energy density. Tesla, Inc., has revealed its plans to gradually increase the use of Si anodes in its future batteries,^7^ and Amprius Technologies, Inc., recently announced the shipment of its first commercially available Si anode based Li-ion batteries with an energy density of 450 mWh g^–1^.^8^

An efficient Si anode remains some sort of *holy grail* for rechargeable Li-ion batteries, and their widespread use is hindered by rapid capacity fading.^9,10^ The enormous volume changes occurring during lithiation/delithiation cycles (e.g., +300% volume increase from Si to Li_22_Si_5_) result in irreversible damage:^4^ deformation and residual stresses accumulate and create an ensemble of structural defect features, including interfaces, dislocations, grain boundaries, and nanocracks forming within the Si anode. An array of approaches has been explored to overcome this critical issue. For example, the use of nanocomposite/structured Si,^11^ including nanowires,^12,13^ core–shell^14,15^ and hollow^16,17^ nanoparticles, and porous Si,^18,19^ has been reported to be effective for the enhanced suppression of the initiation of mechanical fracture from the large volume changes.

Most studies use techniques providing a bulk average or two-dimensional information,^20−22^ which, even in combination, cannot fully analyze the nanoscale compositional distribution and microstructural evolution of electrodes and electrolytes. In situ^11,23,24^ and cryogenic transmission electron microscopy (TEM)^25,26^ have already revealed an undesirable removal or destruction of the passivating solid-electrolyte interphase (SEI) and severe pulverization of Si nano/microparticles from bulk Si during the expansion and shrinkage cycles.^26^ Despite impressive empirical advances, numerous fundamental aspects of the microstructural degradation remain elusive, making it impossible to devise targeted strategies to circumvent these specific issues and enable a breakthrough in Si-based anodes. Elucidating the mechanisms that led to mechanical failure has emerged as a crucial topic to achieve a high-capacity Si anode.

The combination of high-resolution microscopy of the fine scale of the microstructure that develops and the precise microanalysis of the electrode’s evolving composition can be achieved by using the latest development in cryogenic atom probe tomography (cryo-APT), Figure 1a. APT provides direct and three-dimensional, near-atomically resolved analytical imaging of materials and has the ability to collect all elements irrespective of their mass. APT is underpinned by an intense electric field that provides controlled removal of individual ions from a sharp, needle-shaped specimen. However, this field can cause outward electromigration of Li^27^ in battery materials, making the detailed analysis of its distribution often impossible but also affecting the overall data quality,^28^ which explains why battery materials have rarely been analyzed by APT.^29−32^ However, we demonstrated recent approaches enabling analysis of lithiated anode and cathode materials,^28^ and delithiated samples still bear traces of crucial processes taking place during battery operation. Compared with cryo-TEM, the outstanding advantage of cryo-APT is the ability to resolve sub-nm-scale structure and chemistry at the same time and more importantly in three dimensions. Cryo-TEM usually provides a 2D projection of a sometimes complex 3D nanostructure. Tomography can be challenging to interpret from the reconstruction of images acquired through a tilt-series. Chemical information within the sample usually requires the additional incorporation of EELS or EDX with cryo-TEM, which is not always available, and with a sensitivity that is in the range of 1 or more at. % and quantification is particuarly challenging for light elements (e.g., Li). Cryo-APT is however not without challenges, from the difficulties in specimen preparation and transfer to, e.g., the mass ranging with numerous molecular ions and fragmentation paths that can affect their quantification, and this is particularly the case for organic compounds for which the literature is limited.

**Figure 1:**
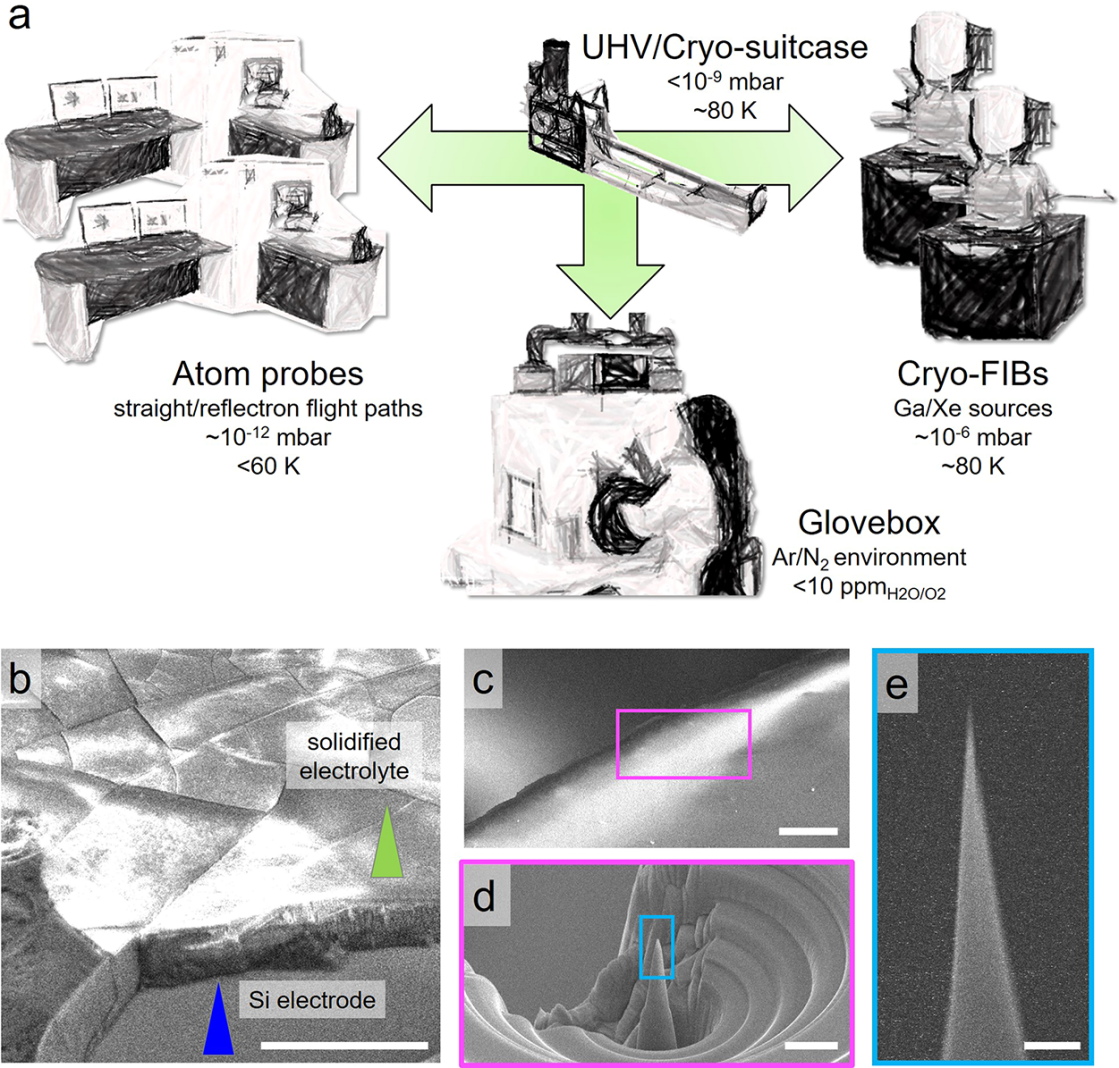
(a) Unique infrastructure for the cryo-atom probe enabling the study. (b) SEM images of the LN2-quenched anode containing the frozen-electrolyte surface. (c) The Si electrode where (d) the cryo-milled pillar was made to prepare (e) the final APT specimen. The scale bars are 50 μm in parts b and c, 20 μm in part d, and 1 μm in part e.

Here, we leverage cryo-APT for the first time to obtain compositional mapping of Li-ion battery materials, the abutting electrolyte, and the solid–liquid interface between the two at an increasing number of charge–discharge cycles. Custom cells were disassembled inside a N_2_ glovebox (H_2_O and O_2_ < 10 ppm);^33^ see the Methods section in the Supporting Information. The collected Si anode with the electrolyte was immediately plunge-frozen in liquid N_2_ (LN_2_) and then transferred by using the cryogenically cooled, ultrahigh-vacuum suitcases into a scanning-electron microscope/Xe-plasma focused ion beam (SEM/PFIB) for imaging and cryogenic specimen preparation, Figure 1b,c. APT specimens of the electrolyte and electrode were prepared at cryogenic temperature using the method we introduced in ref (34), Figure 1d,e.

The locations of the cryo-APT analyses of the uncycled electrode and electrolyte, Figure 2c, are indicatively marked in Figure 1b. Within the electrolyte, individual, isolated Si ions are already detected. We conducted a cryo-APT analysis of the frozen raw electrolyte on a different metallic substrate (NP-Au) that shows no Si ion (see Figures S1–S4). These additional analyses confirm that dissolved Si ions originated from the corrosion of the Si anode. Veith et al. observed non-electrochemically driven Si–O and Si–F bonds on a Si anode soaked in a similar electrolyte.^35^ Si–O groups can react with HF generated by hydrolyzed or thermally decomposed LiPF_6_ electrolyte,^36^ resulting in the dissolution of Si ions and two additional H_2_O molecules, which trigger further HF generation and a self-sustaining corrosive cycle.^37−39^ The hydrolysis could be initiated by residual atmospheric moisture during cell assembly.^40^ Degradation of the anode and the electrolyte hence starts even before cycling, with any oxygen-containing Si species that generate more water and accelerate the failure of the Si battery cell.

**Figure 2:**
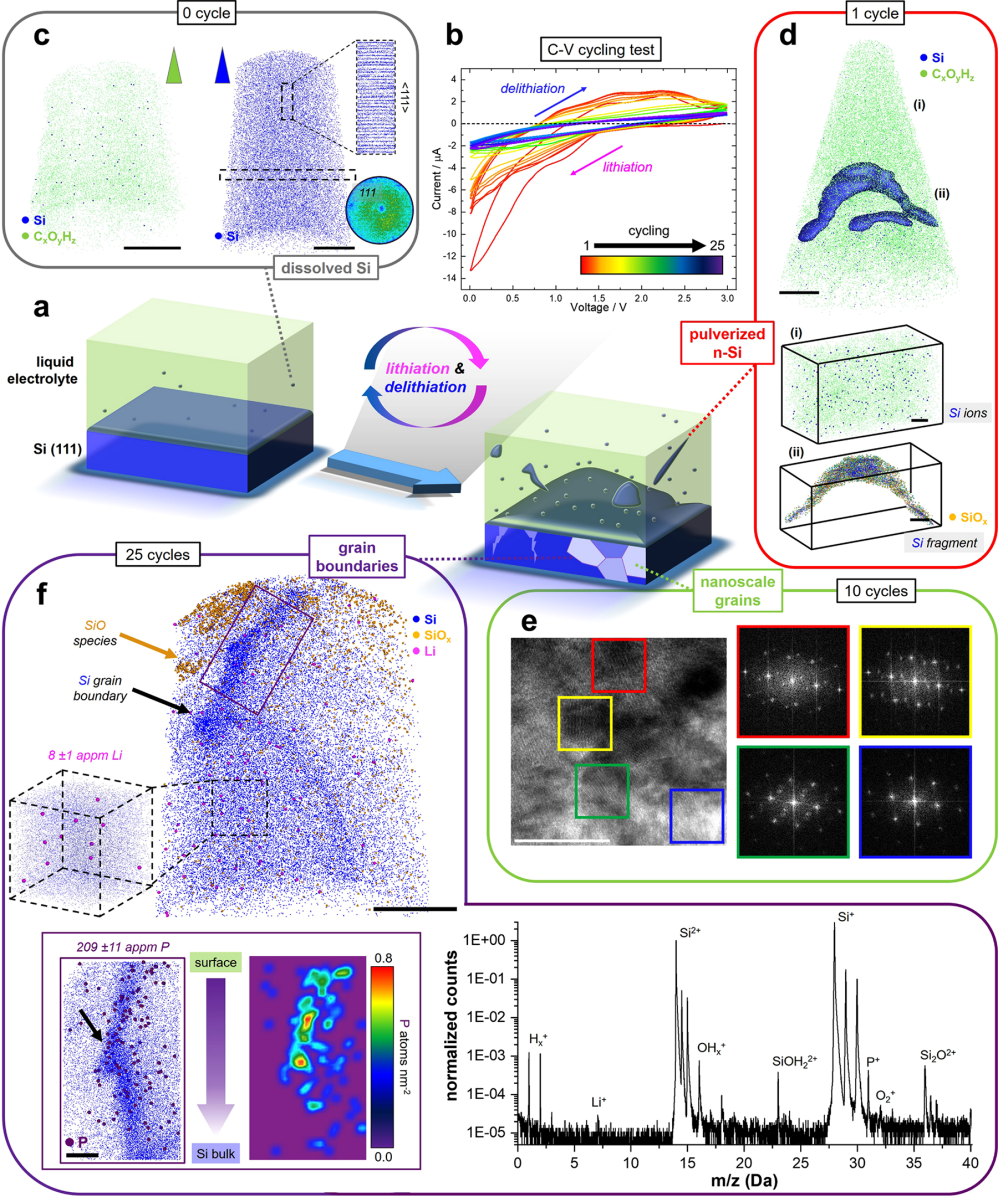
(a) Schematic of the Si electrode and cycling process. (b) Voltage vs current curves of the Si(111) anode in a Li–Si cell. (c) Cryo-APT analysis of the electrolyte and anode before cycling; scale bars are 20 nm. (d) Cryo-APT reconstructed atom map of the one-cycle electrolyte; the blue isosurface delineates regions containing at least 25 at. % Si; the scale bar is 20 nm. Movie #1, the corresponding mass spectra, and additional analyses can be found in the Supporting Information: (i) a close-up showing dissolved Si ions (scale bar = 2 nm) and (ii) a delaminated Si debris in the electrolyte (scale bar = 5 nm). Green, blue, and yellow dots represent reconstructed carbonate species, Si, and SiO_*x*_ compounds, respectively. (e) Transmission-electron micrograph of the 10-cycled Si anode along the [110] zone axis of the single crystal, along with fast Fourier transformation (FFT) patterns from different regions highlighted by colored boxes. The white scale bar is 20 nm. (f) 3D reconstructed atom map of the Si electrode after 25 cycles (scale bar = 20 nm). Blue, yellow, and pink dots represent reconstructed Si, SiO_*x*_, and Li, respectively. Movie #2 and mass spectra of the corresponding data set are presented in the Supporting Information. The inset shows the extracted Si grain boundary with the 2D contour density map of P atoms (scale bar = 5 nm).

After one cycle, Figure 2d, cryo-APT reveals also dissolved isolated Si ions, accompanied by an ∼10 nm pulverized Si fragment covered with an oxide shell (see Figures S5 and S6). Such a fragment could potentially block the pores of the separator for Li-ion diffusion, raising cell impedance and deteriorating the rate performance of the battery. The presence of the SiO_*x*_ species at the surface supports the hypothesis that the dissolution was associated with the formation of HF.

After 10 cycles, TEM was performed on the dried electrode after removal of the electrolyte and thorough cleaning, Figure 2e, complemented by additional APT experiments (Figures S7–S13). We evidence that the originally single crystalline Si has transformed into a nanocrystalline microstructure, containing numerous nanoscale grains and grain boundaries with different crystallographic orientations that have been formed during the lithiation/delithiation process, confirming previous reports.^41^ The volume change associated with the formation of Li-rich phases imposes strong compressive loading on the silicon matrix.^42^ Indentation of Si single crystals has demonstrated that the breaking of covalent Si–Si bonds injects a high number of vacancies in the crystal and results in amorphization,^43,44^ also reported experimentally during battery cycling.^45^ Upon relaxation during delithiation, depending on the rate, new crystals nucleate with no orientation relationship with the surrounding crystal matrix.^43,44^ The discharging rate must influence this process.

After 25 cycles, Figure 2f, cryo-APT analysis of the very surface of the anode contains two Si grains, as confirmed by atom probe crystallography^46^ (Figure S14), and a faceted grain boundary. No chemicals expected from the SEI layer (LiCO_2_, LiOH, LiF) are observed at the interface (see Movie #3), which can be attributed to the high reversibility of the SEI layer on Si anodes.^25,47,48^ On the surface, we find several isolated nanoscale islands rich in SiO_*x*_ species. Such oxides promote the formation of HF which corrodes the Si, passivate the Si anode, and act as a mechanical clamping layer that restricts swelling.^48,49^ SiO_*x*_ can store Li ions (*Q*_SiO_ = 1543 mAh g^–1^)^50^ with lower volume expansion (approximately 120%) when irreversibly lithiated,^51^ that can cause stress build-up at the interface and facilitate crack initiation,^52^ decohesion, and pulverization, explaining the presence of SiO_*x*_ on the Si fragment’s surface in Figure 2d.

After full delithiation, 20–30 nm below the surface, Li (8 ± 1 appm) is still detected within the Si matrix, as readily visible from the corresponding mass spectrum, Figure 2f. Density-functional theory predicts an attraction between vacancies and Li in Si,^53^ which can combine with a strong Coulomb attraction between an electron-rich vacancy and the electropositive Li. The image Li atoms are hence likely trapped by remaining vacancies injected under plastic loading.

At the grain boundary, Li does not appear segregated, conversely to P, that has even seen partitions to specific facets and to the facet junction indicated by a black arrow (see Figure S15). Such a distribution was previously suggested to be associated with local strain.^54^ P can diffuse along grain boundaries in Si,^55^ and its segregation can be energetically favorable due to the passivation of dangling bonds,^56^ which modifies the conductivity.^57^ In addition, atomistic simulations have indicated that the combined effect of the presence of P and a stress concentrator (i.e., a grain boundary) decreases the fracture strength of Si nanowires.^58^ Lastly, the presence of P (209 ± 11 appm), originally from the LiPF_6_ salt, also suggests the liberation of F and the facile formation of HF that is a known embrittler of polycrystalline Si through void formation along grain boundaries.^59^ These effects collectively make these newly created grain boundaries particularly brittle and critical to the lifetime of the Si anode.

To summarize, cryo-APT allowed us to track the evolution of the three-dimensional, nanoscale elemental distributions of species in the electrolyte, a model Si anode, and their interface over increasing charge–discharge cycles. We provide measured data that advance the understanding of the degradation mechanism—or actually degradation mechanisms—and emphasize the often-overlooked role of microstructural defects created and evolving throughout the battery operation lifetime. In addition, the nucleation of the Li_*x*_Si_*y*_ (metastable) phases^42^ during the first cycle can be assumed to be homogeneous, occurring randomly across the surface of the anode. However, the combined presence of crystalline defects and remaining Li impurities in the anode will undoubtedly assist heterogeneous nucleation of these phases during subsequent lithiation, potentially enhanced by accelerated diffusion of Li through structural defects.^60,61^ Segregants can energetically destabilize grain boundaries, already weakened by HF,^59^ or form space charges, that can favor decohesion. Nucleation in the parts of the microstructure with a high density of defects localizes the volume expansion to mechanically weaker regions, thus facilitating the pulverization of fragments from the anode. This combination of (electro)chemical reactions, phase transformation, and mechanical failure, assisted by the localized decomposition of the electrolyte, accelerates the delamination/mass loss and localized lithiation causing fast loss of capacity^51^ (see Figure S16). Strategies for the development of robust and durable Si-based anodes for next-generation Li-ion batteries can draw from our findings on the degradation of the Si electrode—the role of the newly formed grain boundaries that may be exploited through segregation, but also the details of the electrolyte degradation that can guide the selection of P-free and F-free salts and avoiding exposure to moisture during fabrication, which can be difficult to achieve in large-scale production.

## Supporting Information Available

Additional experimental details, materials, methods, cryo-FIB/APT results of the 0-, 1-, and 25-cycled electrolyte/Si anodes, FIB/TEM/APT results of the 10-cycled Si anode, and photographs of the experimental setup and protocols (PDF)
Movie #1 showing the 3D atom map movie of the 25-cycled Si anode (MP4)
Movie #2 showing the 3D atom map movie of the 1-cycled electrolyte (MP4)
Movie #3 showing the 3D atom map movie of the 1-cycled electrolyte/Si anode (MP4)

## References

[ref1] Larcher D.; Tarascon J.-M. Towards Greener and More Sustainable Batteries for Electrical Energy Storage. Nat. Chem. 2015, 7 (1), 19–29. 10.1038/nchem.2085. 25515886

[ref2] Goodenough J. B.; P K.-S. The Li-Ion Rechargeable Battery: A Perspective. J. Am. Chem. Soc. 2013, 135 (4), 1167–1176. 10.1021/ja3091438. 23294028

[ref3] Yan Z.; Tom D.; Maguire G.; da Costa A. N. China EV, Battery Makers Grapple with Graphite Squeeze. Reuters. December 15, 2021. https://www.reuters.com/business/autos-transportation/china-ev-battery-makers-grapple-with-graphite-squeeze-2021-12-15/.

[ref4] McDowell M. T.; Lee S. W.; Nix W. D.; Cui Y. 25th Anniversary Article: Understanding the Lithiation of Silicon and Other Alloying Anodes for Lithium-Ion Batteries. Adv. Mater. 2013, 25 (36), 4966–4985. 10.1002/adma.201301795. 24038172

[ref5] Lai S. Solid Lithium-Silicon Electrode. J. Electrochem. Soc. 1976, 123 (8), 1196–1197. 10.1149/1.2133033.

[ref6] Okamoto H. Li-Si (Lithium-Silicon). J. Phase Equilibria Diffus. 2009, 30 (1), 118–119. 10.1007/s11669-008-9431-8.

[ref7] Tesla, Inc. 2020 Annual Meeting of Stockholders and Battery Day. https://www.tesla.com/en_ca/2020shareholdermeeting.

[ref8] Amprius Technologies, Inc. Amprius Technologies Ships First Commercially Available 450 Wh/kg, 1150 Wh/L Batteries. https://amprius.com/2022/02/amprius-technologies-ships-first-commercially-available-450-wh-kg-1150-wh-l-batteries (accessed Feb 14, 2022).

[ref9] Li H.; Yamaguchi T.; Matsumoto S.; Hoshikawa H.; Kumagai T.; Okamoto N. L.; Ichitsubo T. Circumventing Huge Volume Strain in Alloy Anodes of Lithium Batteries. Nat. Commun. 2020, 11 (1), 1584 10.1038/s41467-020-15452-0. 32284535 PMC7154030

[ref10] Obrovac M. N.; Chevrier V. L. Alloy Negative Electrodes for Li-Ion Batteries. Chem. Rev. 2014, 114 (23), 11444–11502. 10.1021/cr500207g. 25399614

[ref11] Liu X. H.; Zhong L.; Huang S.; Mao S. X.; Zhu T.; Huang J. Y. Size-Dependent Fracture of Silicon Nanoparticles During Lithiation. ACS Nano 2012, 6 (2), 1522–1531. 10.1021/nn204476h. 22217200

[ref12] Chan C. K.; Patel R. N.; O’Connell M. J.; Korgel B. A.; Cui Y. Solution-Grown Silicon Nanowires for Lithium-Ion Battery Anodes. ACS Nano 2010, 4 (3), 1443–1450. 10.1021/nn901409q. 20201547

[ref13] Chan C. K.; Peng H.; Liu G.; McIlwrath K.; Zhang X. F.; Huggins R. A.; Cui Y. High-Performance Lithium Battery Anodes Using Silicon Nanowires. Nat. Nanotechnol. 2008, 3 (1), 31–35. 10.1038/nnano.2007.411. 18654447

[ref14] Nava G.; Schwan J.; Boebinger M. G.; McDowell M. T.; Mangolini L. Silicon-Core–Carbon-Shell Nanoparticles for Lithium-Ion Batteries: Rational Comparison between Amorphous and Graphitic Carbon Coatings. Nano Lett. 2019, 19 (10), 7236–7245. 10.1021/acs.nanolett.9b02835. 31539476

[ref15] Zhang T.; Gao J.; Zhang H. P.; Yang L. C.; Wu Y. P.; Wu H. Q. Preparation and Electrochemical Properties of Core-Shell Si/SiO Nanocomposite as Anode Material for Lithium Ion Batteries. Electrochem. commun. 2007, 9 (5), 886–890. 10.1016/j.elecom.2006.11.026.

[ref16] Yao Y.; McDowell M. T.; Ryu I.; Wu H.; Liu N.; Hu L.; Nix W. D.; Cui Y. Interconnected Silicon Hollow Nanospheres for Lithium-Ion Battery Anodes with Long Cycle Life. Nano Lett. 2011, 11 (7), 2949–2954. 10.1021/nl201470j. 21668030

[ref17] Chen Y.; Hu Y.; Shen Z.; Chen R.; He X.; Zhang X.; Li Y.; Wu K. Hollow Core–Shell Structured Silicon@carbon Nanoparticles Embed in Carbon Nanofibers as Binder-Free Anodes for Lithium-Ion Batteries. J. Power Sources 2017, 342, 467–475. 10.1016/j.jpowsour.2016.12.089.

[ref18] Ge M.; Rong J.; Fang X.; Zhou C. Porous Doped Silicon Nanowires for Lithium Ion Battery Anode with Long Cycle Life. Nano Lett. 2012, 12 (5), 2318–2323. 10.1021/nl300206e. 22486769

[ref19] Xiao J.; Xu W.; Wang D.; Choi D.; Wang W.; Li X.; Graff G. L.; Liu J.; Zhang J.-G. Stabilization of Silicon Anode for Li-Ion Batteries. J. Electrochem. Soc. 2010, 157 (10), A1047 10.1149/1.3464767.

[ref20] Schellenberger M.; Golnak R.; Quevedo Garzon W. G.; Risse S.; Seidel R. Accessing the Solid Electrolyte Interphase on Silicon Anodes for Lithium-Ion Batteries in-Situ through Transmission Soft X-Ray Absorption Spectroscopy. Mater. Today Adv. 2022, 14, 100215 10.1016/j.mtadv.2022.100215.

[ref21] Wang L.; Menakath A.; Han F.; Wang Y.; Zavalij P. Y.; Gaskell K. J.; Borodin O.; Iuga D.; Brown S. P.; Wang C.; et al. Identifying the Components of the Solid–Electrolyte Interphase in Li-Ion Batteries. Nat. Chem. 2019, 11 (9), 789–796. 10.1038/s41557-019-0304-z. 31427766

[ref22] Chen C.; Zhou T.; Danilov D. L.; Gao L.; Benning S.; Schön N.; Tardif S.; Simons H.; Hausen F.; Schülli T. U.; et al. Impact of Dual-Layer Solid-Electrolyte Interphase Inhomogeneities on Early-Stage Defect Formation in Si Electrodes. Nat. Commun. 2020, 11 (1), 5074 10.1038/s41467-020-18895-7. 32612261 PMC7329811

[ref23] McDowell M. T.; Lee S. W.; Harris J. T.; Korgel B. A.; Wang C.; Nix W. D.; Cui Y. In Situ TEM of Two-Phase Lithiation of Amorphous Silicon Nanospheres. Nano Lett. 2013, 13 (2), 758–764. 10.1021/nl3044508. 23323680

[ref24] McDowell M. T.; Ryu I.; Lee S. W.; Wang C.; Nix W. D.; Cui Y. Studying the Kinetics of Crystalline Silicon Nanoparticle Lithiation with In Situ Transmission Electron Microscopy. Adv. Mater. 2012, 24 (45), 6034–6041. 10.1002/adma.201202744. 22945804

[ref25] Huang W.; Wang J.; Braun M. R.; Zhang Z.; Li Y.; Boyle D. T.; McIntyre P. C.; Cui Y. Dynamic Structure and Chemistry of the Silicon Solid-Electrolyte Interphase Visualized by Cryogenic Electron Microscopy. Matter 2019, 1 (5), 1232–1245. 10.1016/j.matt.2019.09.020.

[ref26] He Y.; Jiang L.; Chen T.; Xu Y.; Jia H.; Yi R.; Xue D.; Song M.; Genc A.; Bouchet-Marquis C.; et al. Progressive Growth of the Solid–Electrolyte Interphase towards the Si Anode Interior Causes Capacity Fading. Nat. Nanotechnol. 2021, 16 (10), 1113–1120. 10.1038/s41565-021-00947-8. 34326526

[ref27] Pfeiffer B.; Maier J.; Arlt J.; Nowak C. In Situ Atom Probe Deintercalation of Lithium-Manganese-Oxide. Microsc. Microanal. 2017, 23 (2), 314–320. 10.1017/S1431927616012691. 28134068

[ref28] Kim S.-H.; Antonov S.; Zhou X.; Stephenson L. T.; Jung C.; El-Zoka A. A.; Schreiber D. K.; Conroy M.; Gault B. Atom Probe Analysis of Electrode Materials for Li-Ion Batteries: Challenges and Ways Forward. J. Mater. Chem. A 2022, 10, 4926–4935. 10.1039/D1TA10050E. PMC8887568 35341092

[ref29] Devaraj A.; Gu M.; Colby R.; Yan P.; Wang C. M.; Zheng J. M.; Xiao J.; Genc A.; Zhang J. G.; Belharouak I. Visualizing Nanoscale 3D Compositional Fluctuation of Lithium in Advanced Lithium-Ion Battery Cathodes. Nat. Commun. 2015, 6, 8014 10.1038/ncomms9014. 26272722 PMC4557343

[ref30] Mohanty D.; Mazumder B.; Devaraj A.; Sefat A. S.; Huq A.; David L. A.; Payzant E. A.; Li J.; Wood D. L.; Daniel C. Resolving the Degradation Pathways in High-Voltage Oxides for High-Energy-Density Lithium-Ion Batteries; Alternation in Chemistry, Composition and Crystal Structures. Nano Energy 2017, 36, 76–84. 10.1016/j.nanoen.2017.04.008.

[ref31] Chae B.-G.; Park S. Y.; Song J. H.; Lee E.; Jeon W. S. Evolution and Expansion of Li Concentration Gradient during Charge–Discharge Cycling. Nat. Commun. 2021, 12 (1), 3814 10.1038/s41467-021-24120-w. 34155217 PMC8217543

[ref32] Maier J.; Pfeiffer B.; Volkert C. A.; Nowak C. Three-Dimensional Microstructural Characterization of Lithium Manganese Oxide with Atom Probe Tomography. Energy Technol. 2016, 4 (12), 1565–1574. 10.1002/ente.201600210.

[ref33] Stephenson L. T.; Szczepaniak A.; Mouton I.; Kristiane A.; Rusitzka K.; Breen A. J.; Tezins U.; Sturm A.; Vogel D.; Chang Y.; et al. The Laplace Project: An Integrated Suite for Preparing and Transferring Atom Probe Samples under Cryogenic and UHV Conditions. PLoS One 2018, 13, e0209211 10.1371/journal.pone.0209211. 30576351 PMC6303089

[ref34] El-Zoka A. A.; Kim S.-H.; Deville S.; Newman R. C.; Stephenson L. T.; Gault B. Enabling Near-Atomic–Scale Analysis of Frozen Water. Sci. Adv. 2020, 6 (49), eabd6324 10.1126/sciadv.abd6324. 33277259 PMC7821902

[ref35] Veith G. M.; Baggetto L.; Sacci R. L.; Unocic R. R.; Tenhaeff W. E.; Browning J. F. Direct Measurement of the Chemical Reactivity of Silicon Electrodes with LiPF6-Based Battery Electrolytes. Chem. Commun. 2014, 50 (23), 3081–3084. 10.1039/c3cc49269a. 24513965

[ref36] McBrayer J. D.; Rodrigues M.-T. F.; Schulze M. C.; Abraham D. P.; Apblett C. A.; Bloom I.; Carroll G. M.; Colclasure A. M.; Fang C.; Harrison K. L.; et al. Calendar Aging of Silicon-Containing Batteries. Nat. Energy 2021, 6 (9), 866–872. 10.1038/s41560-021-00883-w.

[ref37] Lehmann V. The Chemical Dissolution of Silicon. Electrochemistry of Silicon 2002, 23–38. 10.1002/3527600272.ch2.

[ref38] Saqib N.; Ganim C. M.; Shelton A. E.; Porter J. M. On the Decomposition of Carbonate-Based Lithium-Ion Battery Electrolytes Studied Using Operando Infrared Spectroscopy. J. Electrochem. Soc. 2018, 165 (16), A4051–A4057. 10.1149/2.1051816jes.

[ref39] Agubra V. A.; Fergus J. W. The Formation and Stability of the Solid Electrolyte Interface on the Graphite Anode. J. Power Sources 2014, 268, 153–162. 10.1016/j.jpowsour.2014.06.024.

[ref40] Wiemers-Meyer S.; Jeremias S.; Winter M.; Nowak S. Influence of Battery Cell Components and Water on the Thermal and Chemical Stability of LiPF6 Based Lithium Ion Battery Electrolytes. Electrochim. Acta 2016, 222, 1267–1271. 10.1016/j.electacta.2016.11.100.

[ref41] Shi F.; Song Z.; Ross P. N.; Somorjai G. A.; Ritchie R. O.; Komvopoulos K. Failure Mechanisms of Single-Crystal Silicon Electrodes in Lithium-Ion Batteries. Nat. Commun. 2016, 7 (1), 11886 10.1038/ncomms11886. 27297565 PMC4911629

[ref42] Chan M. K. Y.; Wolverton C.; Greeley J. P. First Principles Simulations of the Electrochemical Lithiation and Delithiation of Faceted Crystalline Silicon. J. Am. Chem. Soc. 2012, 134 (35), 14362–14374. 10.1021/ja301766z. 22817384

[ref43] Ruffell S.; Bradby J. E.; Williams J. S. High Pressure Crystalline Phase Formation during Nanoindentation: Amorphous versus Crystalline Silicon. Appl. Phys. Lett. 2006, 89 (9), 091919 10.1063/1.2339039.

[ref44] Vandeperre L. J.; Giuliani F.; Lloyd S. J.; Clegg W. J. The Hardness of Silicon and Germanium. Acta Mater. 2007, 55 (18), 6307–6315. 10.1016/j.actamat.2007.07.036.

[ref45] Mozhzhukhina N.; Flores E.; Lundström R.; Nyström V.; Kitz P. G.; Edström K.; Berg E. J. Direct Operando Observation of Double Layer Charging and Early Solid Electrolyte Interphase Formation in Li-Ion Battery Electrolytes. J. Phys. Chem. Lett. 2020, 11 (10), 4119–4123. 10.1021/acs.jpclett.0c01089. 32354215 PMC7467741

[ref46] Gault B.; Moody M. P.; Cairney J. M.; Ringer S. P. Atom Probe Crystallography. Mater. Today 2012, 15, 378 10.1016/S1369-7021(12)70164-5.

[ref47] Zhang X.; Weng S.; Yang G.; Li Y.; Li H.; Su D.; Gu L.; Wang Z.; Wang X.; Chen L. Interplay between Solid-Electrolyte Interphase and (in)Active LixSi in Silicon Anode. Cell Reports Phys. Sci. 2021, 2 (12), 100668 10.1016/j.xcrp.2021.100668.

[ref48] Wu H.; Chan G.; Choi J. W.; Ryu I.; Yao Y.; McDowell M. T.; Lee S. W.; Jackson A.; Yang Y.; Hu L.; et al. Stable Cycling of Double-Walled Silicon Nanotube Battery Anodes through Solid–Electrolyte Interphase Control. Nat. Nanotechnol. 2012, 7 (5), 310–315. 10.1038/nnano.2012.35. 22447161

[ref49] Sivonxay E.; Aykol M.; Persson K. A. The Lithiation Process and Li Diffusion in Amorphous SiO2 and Si from First-Principles. Electrochim. Acta 2020, 331, 135344 10.1016/j.electacta.2019.135344.

[ref50] Zhao J.; Lee H.-W.; Sun J.; Yan K.; Liu Y.; Liu W.; Lu Z.; Lin D.; Zhou G.; Cui Y. Metallurgically Lithiated SiO&lt;Sub&gt;X&lt;/Sub&gt; Anode with High Capacity and Ambient Air Compatibility. Proc. Natl. Acad. Sci. U. S. A. 2016, 113 (27), 7408–7413. 10.1073/pnas.1603810113. 27313206 PMC4941422

[ref51] Pan K.; Zou F.; Canova M.; Zhu Y.; Kim J.-H. Systematic Electrochemical Characterizations of Si and SiO Anodes for High-Capacity Li-Ion Batteries. J. Power Sources 2019, 413, 20–28. 10.1016/j.jpowsour.2018.12.010.

[ref52] Kontis P.; Li Z.; Segersäll M.; Moverare J. J. J. J.; Reed R. C. C. R. C.; Raabe D.; Gault B. The Role of Oxidized Carbides on Thermal-Mechanical Performance of Polycrystalline Superalloys. Metall. Mater. Trans. A 2018, 49 (9), 4236–4245. 10.1007/s11661-018-4709-x.

[ref53] Huang J.; Wang Z.; Gong X.; Wu M.; Liu G.; Lei X.; Liang J.; Cao H.; Tang F.; Lei M.; et al. Vacancy Assisted Li Intercalation in Crystalline Si as Anode Materials for Lithium Ion Batteries. Int. J. Electrochem. Sci. 2013, 8, 5643–5649.

[ref54] Liebscher C. H.; Stoffers A.; Alam M.; Lymperakis L.; Cojocaru-Mirédin O.; Gault B.; Neugebauer J.; Dehm G.; Scheu C.; Raabe D. Strain-Induced Asymmetric Line Segregation at Faceted Si Grain Boundaries. Phys. Rev. Lett. 2018, 121 (1), 15702 10.1103/PhysRevLett.121.015702. 30028158

[ref55] Holloway P. H. Grain Boundary Diffusion of Phosphorus in Polycrystalline Silicon. J. Vac. Sci. Technol. 1982, 21 (1), 19–22. 10.1116/1.571713.

[ref56] Zhao D.; Li Y. Revealing the Factors Influencing Grain Boundary Segregation of P, As in Si: Insights from First-Principles. Acta Mater. 2019, 168, 52–62. 10.1016/j.actamat.2019.02.014.

[ref57] Carabelas A.; Nobili D.; Solmi S. GRAIN BOUNDARY SEGREGATION IN SILICON HEAVILY DOPED WITH PHOSPHORUS AND ARSENIC. J. Phys. Colloq. 1982, 43 (C1), C1-187–C1-192. 10.1051/jphyscol:1982125.

[ref58] Liu B.; Tao J. Y.; Chen X.; Zhang Y. A.; Jiang Y.; Qian Y. Numerical Investigation of the Effects of Phosphorus on the Mechanical Responses of [1 1 0]-Oriented Silicon Nano-Wires. Microelectron. Reliab. 2016, 64, 225–229. 10.1016/j.microrel.2016.07.070.

[ref59] Kageyama Y.; Murase Y.; Tsuchiya T.; Funabashi H.; Sakata J. Formation of Porous Grain Boundaries in Polycrystalline Silicon Thin Films. J. Appl. Phys. 2002, 91 (11), 9408 10.1063/1.1476088.

[ref60] Peng Z.; Meiners T.; Lu Y.; Liebscher C. H.; Kostka A.; Raabe D.; Gault B. Quantitative Analysis of Grain Boundary Diffusion, Segregation and Precipitation at a Sub-Nanometer Scale. Acta Mater. 2022, 225, 117522 10.1016/j.actamat.2021.117522.

[ref61] Legros M.; Dehm G.; Arzt E.; Balk T. J. J. Observation of Giant Diffusivity Along Dislocation Cores. Science (80-.) 2008, 319 (5870), 1646–1649. 10.1126/science.1151771. 18356520

