# Supplementary material for: Understanding the Degradation of a Model Si Anode in a Li-Ion Battery at the Atomic Scale
Source: J Phys Chem Lett. 2022 Sep 1;13(36):8416–21. doi: 10.1021/acs.jpclett.2c02236 (PMC9486947; doi:10.1021/acs.jpclett.2c02236)
Supplement: Supplementary file 1 — jz2c02236_si_001.pdf [file jz2c02236_si_001.pdf]

Supplementary Information

Understanding the Degradation of a Model Si-anode in Li-ion Battery at the Atomic-scale

Authors: Se-Ho Kim[1,†,*], Kang Dong[2,†], Huan Zhao[1], Ayman A. El-Zoka[1], Xuyang Zhou[1], Eric V. Woods[1], Finn Giuliani[3], Ingo Manke[2], Dierk Raabe[1], Baptiste Gault[1,3,*]

Affiliations:
[1]Max-Planck-Institut für Eisenforschung GmbH, Max-Planck-Straße 1, 40237 Düsseldorf, Germany

[2]Institute of Applied Materials, Helmholtz-Zentrum Berlin für Materialien and Energie, 14109 Berlin, Germany

[3]Department of Materials, Royal School of Mines, Imperial College, SW7 2AZ London, United Kingdom

[†]co-first authors

[*]corresponding authors

Methods

Materials

Silicon wafer (0.5 mm thick, no dopant, (111)) and an electrolyte of 1 M $LiPF_6$ with a mixture of ethylene carbonate (EC) and diethyl carbonate (DEC) (1:1, v/v) were received from Sigma-Aldrich. Metallic lithium was purchased from MTI Corp. USA. A Swagelok derived cell consisting of a polyether ether ketone (PEEK) housing is described in our previous report[1].

Battery Assembly and Cycling

Si wafer was cut into 3.0 mm disks by laser-cutting under Argon gas protection. The obtained Si disks were washed using ultrapure water (Milli-Q) and 2-propanol (HPLC Plus, 99.9%, Sigma-Aldrich) to remove contaminations (*e.g.* dust) on the surface from the cutting process. Li chips were punched into disks with a diameter of 3 mm as the counter electrode without further treatment. To preserve the SEI layer on the surface of Si wafer, instead of using a typical Celgard or glassfiber separator, we opt for a ring-shaped spacer (0.5 mm thick) made of polytetrafluoroethylene (PTFE) as a separator. In this way, the Si surface including the SEI layer at the middle area of the Si disk could keep intact without undergoing a peeling-off procedure during the cell disassembly. The Li/Si cells were built using the customized Swagelok-type cell in an argon-filled MBraun glovebox (H_2O and O_2 < 5 ppm). After cell assembly, Li/Si cells were subjected to cyclic voltammetry (CV) cycling between 0.01 – 3.0 V at a scan rate of 0.2 mV/s using a BioLogic MPG-200 potentiostat. All the cells were stopped after 0, 1, 10, 25 cycles at the delithiation state (see Fig. S17).

Sample preparation

After cycling, the cell was disassembled in a nitrogen filled glove-box (<10 ppm H_2O and O_2). Without washing, the Si disk was mounted on a Cu clip and was rapidly plunged into liquid nitrogen, followed by loading on a scanning-electron microscope/Xe-plasma focused ion beam (SEM/p-FIB) (Helios PFIB, Thermo-Fisher, Eindhoven, Netherlands) stage. Subsequently, the Si disk was transferred into the cryo-p-FIB chamber using the ultra-high vacuum transfer suitcase (10^{-9} mbar, -190 ^oC) (VSN-40, Ferrovac GmbH, Zurich, Switzerland) to avoid the sample exposure to air. An illustration of environmentally sensitive sample preparation/transfer for FIB/APT is shown in Fig. S18.

Cryo-APT specimen preparation

The cryo-p-FIB stage (Gatan C1001, Gatan Inc., California, USA) was pre-cooled to -190 ^oC by cold N_2 gas. A clean pillar from frozen electrolyte and Si anode was prepared using the in-situ non lift-out protocol described in references[2,3]. After the height of the post had reached 50 µm, progressively the frozen sample were sharpened into APT specimen using annular milling patterns (e.g. specimen-radius less than 100 nm). Scanning electron

micrographs were taken at 5-15 kV and 1.6-2.3 nA to avoid charging effects and the e-beam-induced diffusion/reaction (see Fig. S19 and S20).

APT measurement

Atom probe data were acquired from 5000 XS instrument (CAMECA, Madison, USA) in pulsed laser mode with laser energy of 20-40 pJ for the cold Si anode (80 pJ for the frozen electrolytes) and rate of 100 kHz at 1 % detection rate. The base temperature was set to 60 K throughout the measurement and the applied direct current was adjusted to control the stable evaporation (see Fig. S21). The atom map reconstruction and data analysis were done using AP SUITE 6.1 software developed by CAMECA.

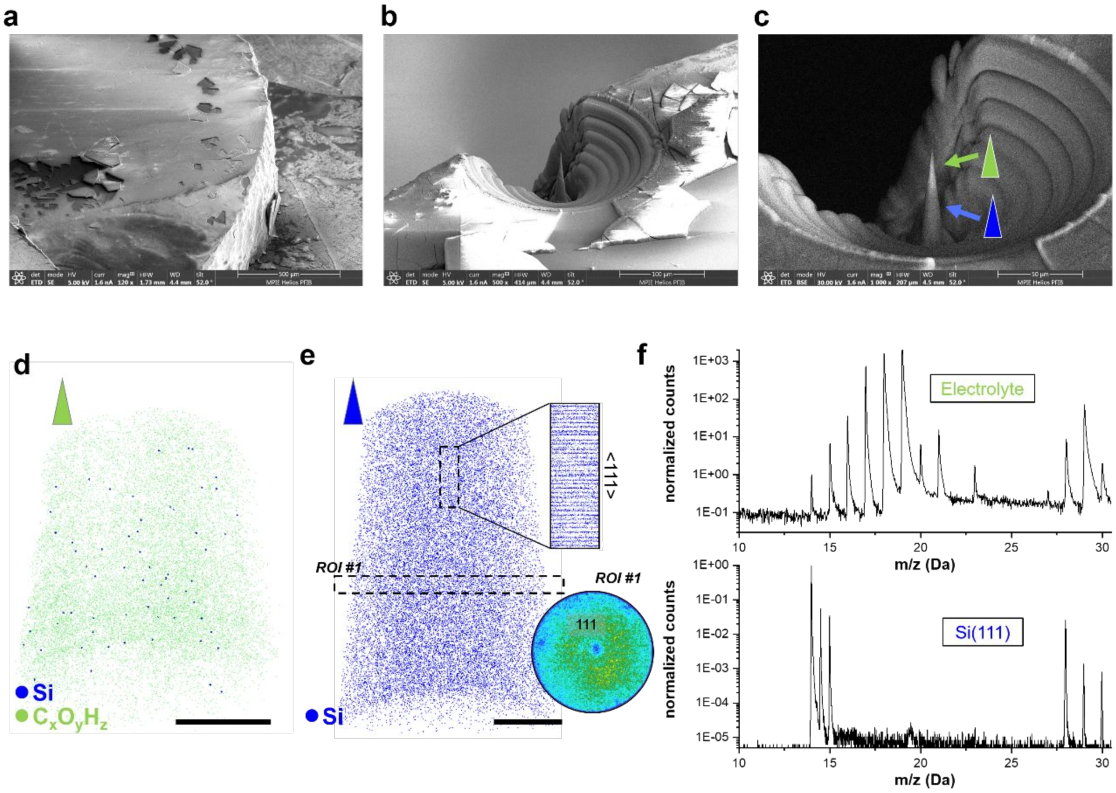

Fig. S1. (a) SEM image of frozen 0-cycle Si anode. (b) In-situ annular milling process. (c) Final APT specimens. 3D atom maps of the 0-cycle specimen: (d) electrolyte and (e) single crystal Si(111) anode. Scale bars are 20 nm. (f) corresponding mass spectrum of each. Here in the electrolyte mass-spectrum, no Li peaks were measured; however, we detected a strong peak at 19-21 Da which could be originated from LiC^+ (or F^+) and $LiCH_x^+$ (or H_xF/LiO_x^+) species. Nevertheless, herein, no segregation behavior of carbonate species nor no $LiPF_6$ salt are detected. The decomposed C:O atomic ratio from the acquired mass spectrum is 1.07 which supports that the frozen specimen is the electrolyte compound. The molecular formulae of mixed organic solvent of ethyl carbonate (EC) and dimethyl carbonate (DMC) compounds are $C_3O_3H_4$ and $C_3O_3H_6$, respectively, so it is difficult to conclude which molecules they are. Apart from that, as expected, the atom map of the as-received single-crystal Si anode shows [111] crystallographic pole at the center with Si(111) atomic planes readily visible.

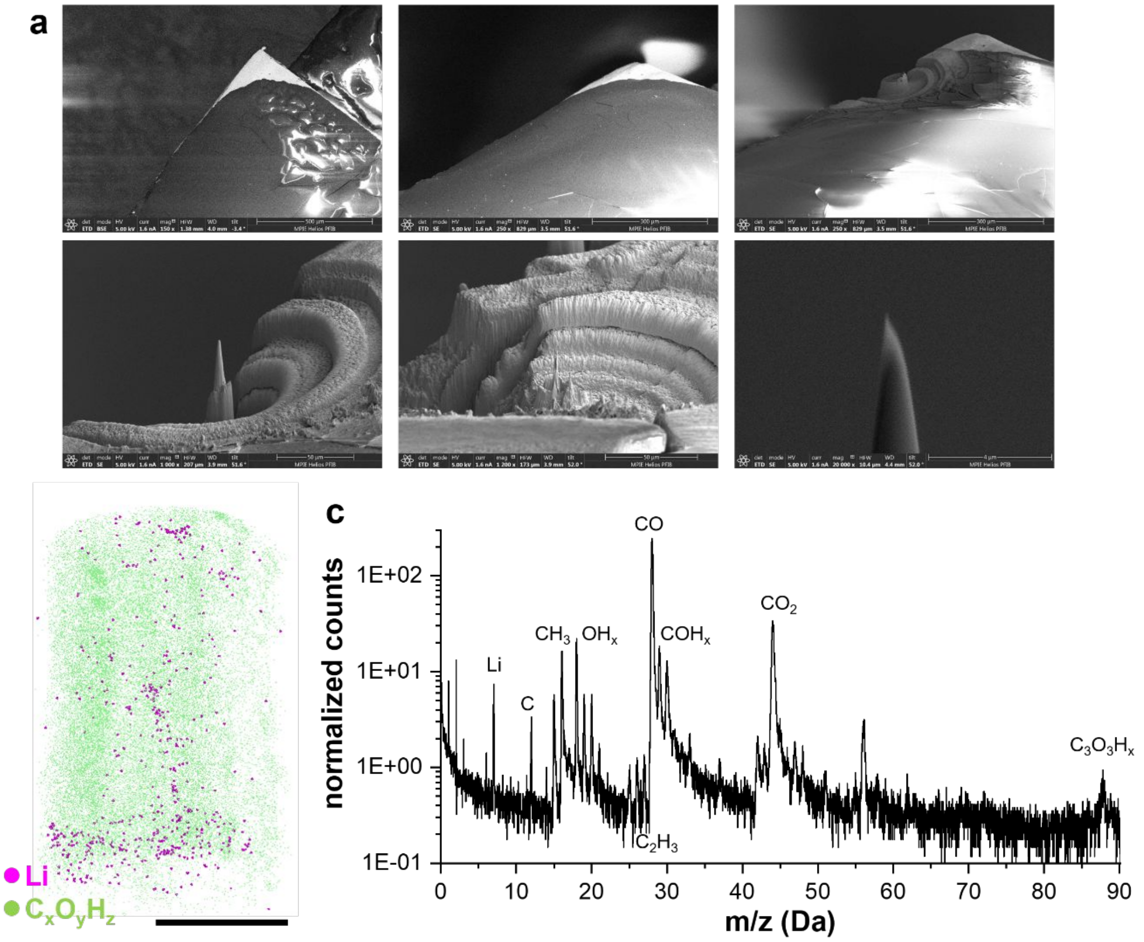

Fig. S2. (a) Cryo-APT specimen preparation from a raw electrolyte (non-contact with Si). (b) 3D atom map of a raw electrolyte (a scale bar = 20 nm) and (c) corresponding mass spectrum. Note that there are no Si peaks. Nano-porous gold instead of Si was used as a substrate to hold the frozen raw liquid electrolyte for cryo-APT measurement. The reconstructed Li ions (6,7 Da) are segregated locally implying that there was a phase separation of the Li salt during freezing. In the mass spectrum of the pristine (non-Si contacted) electrolyte, notable peaks at 90-85 Da are measured which originates to the $C_3O_3H_x^+$ molecular species.

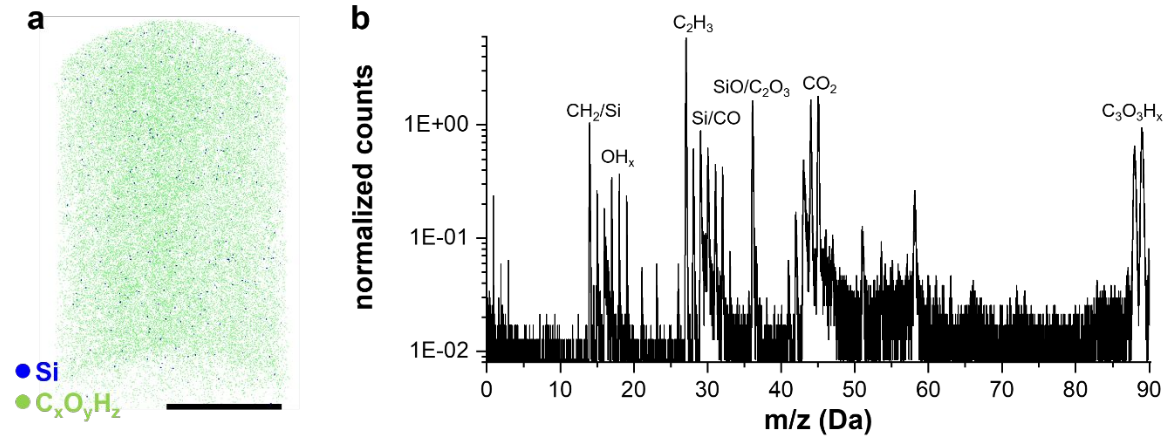

Fig. S3. Additional cryo-APT measurement of the 1-cycle electrolyte. (a) 3D atom map of 1-cycle electrolyte and (b) corresponding mass spectrum. Note that there is Si peaks. A scale bar is 20 nm.

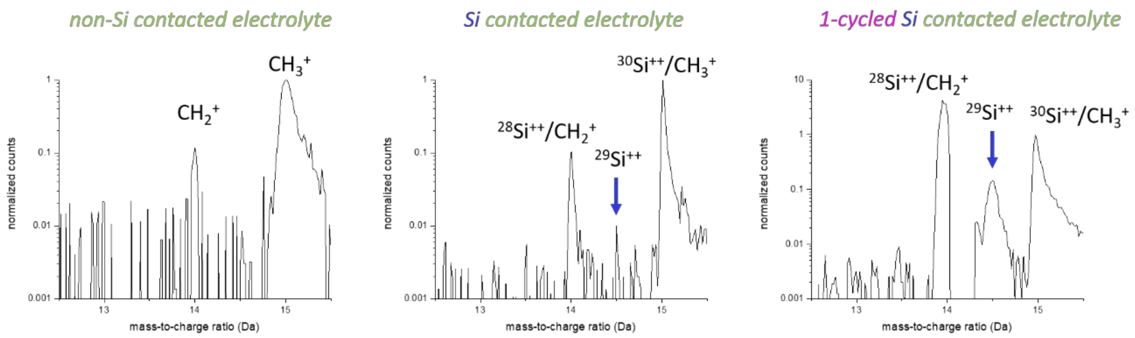

Fig. S4. Background corrected mass spectra of non-Si, Si-contacted and 1-cycled electrolyte. Note that peaks at 14.5 Da aren't likely from CO^{++} state. The second ionization energy of CO requires 41.8 ±0.5 eV (for CO^+ = 14.07 ±0.05 eV)[4] whereas ionization energies of Si^+ and Si^{++} are 8.15 and 16.34 eV. Therefore, detecting CO^{++} is extremely unlikely.

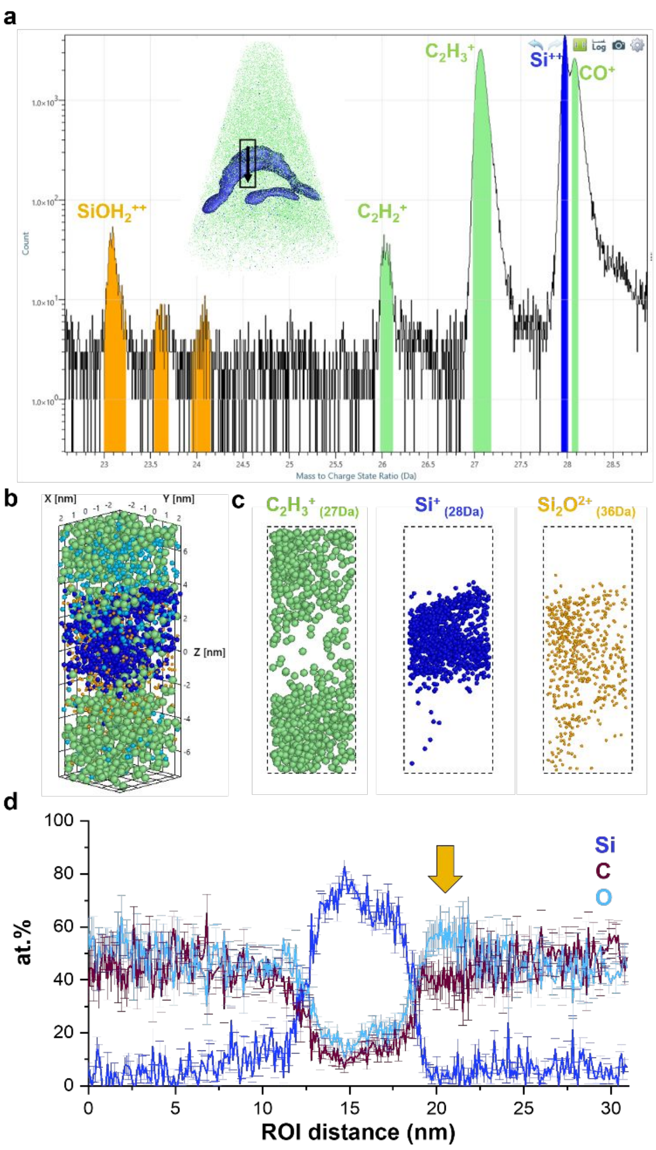

Fig. S5. APT analysis of 1-cycled electrolyte sample. (a) Mass spectrum of cycled electrolyte APT dataset. Note that there is a strong peak split at 28 Da originated to overlapped peaks of CO^+ and Si^+ ions, which is commonly seen in the CO measurement[5]. Inset shows that corresponding 3D atom map. (b) Extracted region of interest ($5\times5\times15$ nm^3) from Fig. 2d. (c) ions distributions: $C_2H_3^+$(green), Si^+(blue), and Si_2O^+ (orange). (d) 1D compositional profiles along the Si nano-fragment in the measurement direction. Note that O enrichment at the interface can be observed (orange arrow).

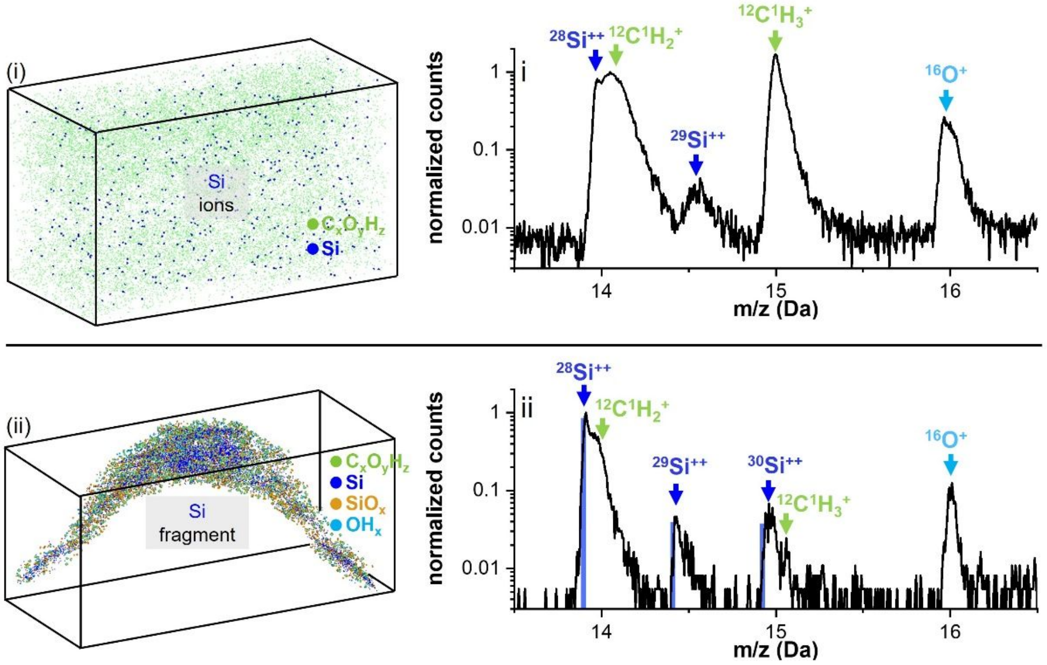

Fig. S6. Mass spectrum of each extracted volume from 1-cycled electrolyte APT dataset (Fig. 2d). Blue lines on the mass spectrum indicate a ratio of natural abundance isotopes of silicon.

APT specimen and TEM lamella preparation from the 10-cycled Si anode

After the cycling for 10 times, the Si anode was collected and rinsed with 1-methyl-2-pyrrolidone (NMP) (anhydrous 95%, Sigma Aldrich) solvent inside the N_2 glovebox. After removing surface residuals, it was dried in a vacuum chamber attached to the glovebox for 1 hr. Subsequently, the sample was loaded to the precision etching coating system (PECS) II (Model 685, Gatan). A 50 nm layer of Cr was deposited on the sample for surface protection. The coated sample was loaded to the dual-beam FIB (FEI Helios Nanolab 600) chamber. APT specimens from the 10-cycled sample were prepared using Ga-ion milling according to ref.[6]. Three distinguishable interest regions (topmost surface, near-surface, and bulk) were fabricated into the APT specimens. For TEM lamella, first, the surface was coated additionally with e- and Ga-ion beam-induced Pt/C layer. Then the lamella was obtained mostly following the protocol described in ref.[7].

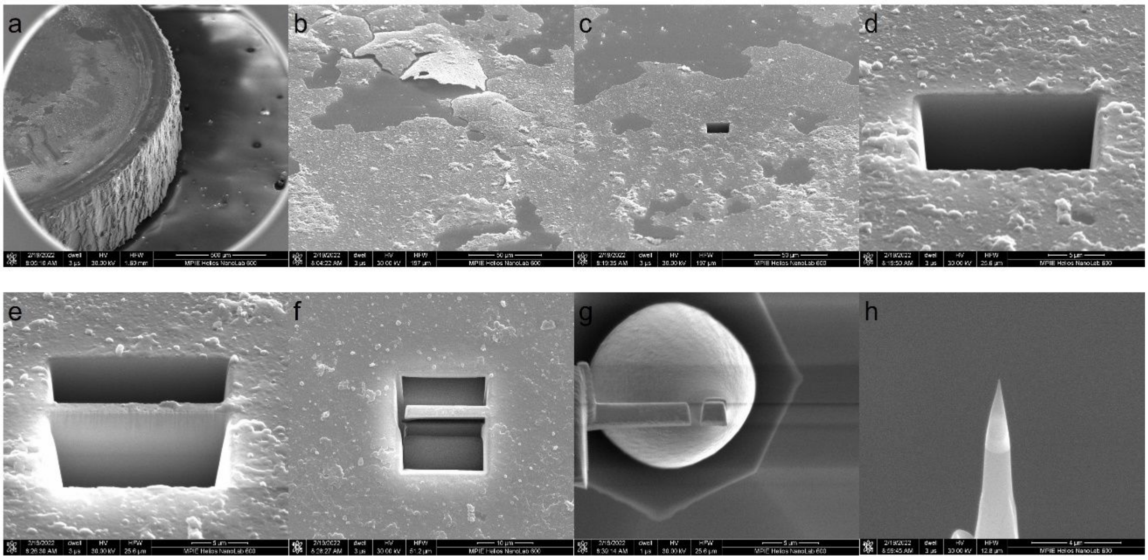

Fig. S7. APT specimen preparation of the 10-cycle Si anode. The disassembled electrode was coated with PECs-Cr layer (~50 nm). (a) 52°-tilted Si anode. (b) FIB/SEM surface image shows that there are delaminated layers of residuals (*i.e.* salt (please see APT/TEM results in S9 and 11)). (c)-(e) Front and back-side cuts. (f) L-shape cut to free the lamella. (g) Mounted APT sample on a commercial Si micro-post. (h) A final APT specimen.

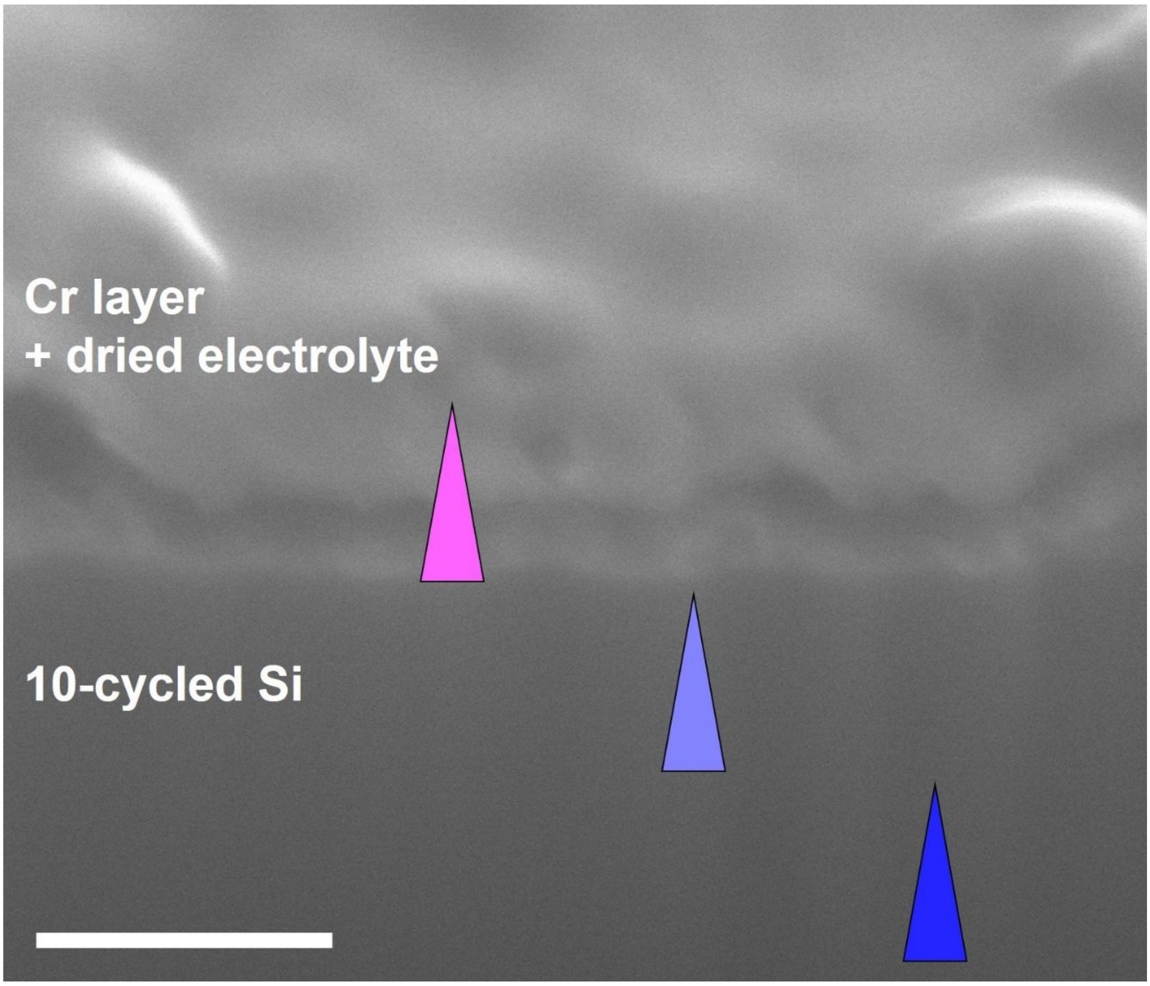

Fig. S8. A cross-sectional FIB/SEM image of the 10-cycled Si anode (scale bar = 500 nm). The colored triangles indicate the regions of three representative APT measurements that are presented in Fig. S9.

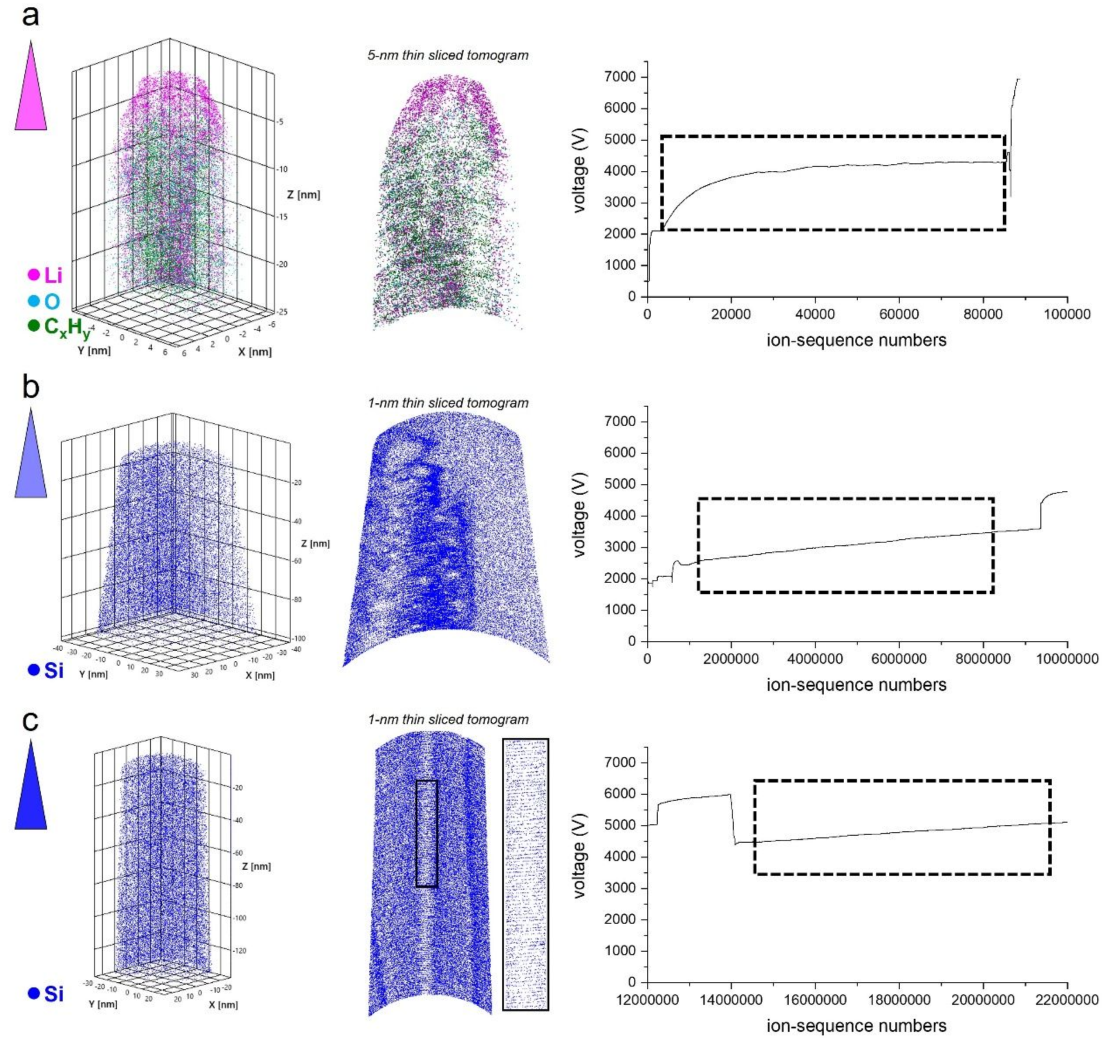

Fig. S9. APT analysis (3D atom map, tomogram, and V-curves) of (a) salt layer, (b) near-surface Si, (c) bottom Si from the 10-cycled Si sample. Note that ambiguous peaks in the APT dataset from the salt layer were not ranged, for instance F (LiC) *vs.* H_3O or Li_2C *vs.* C_2H_2 etc.

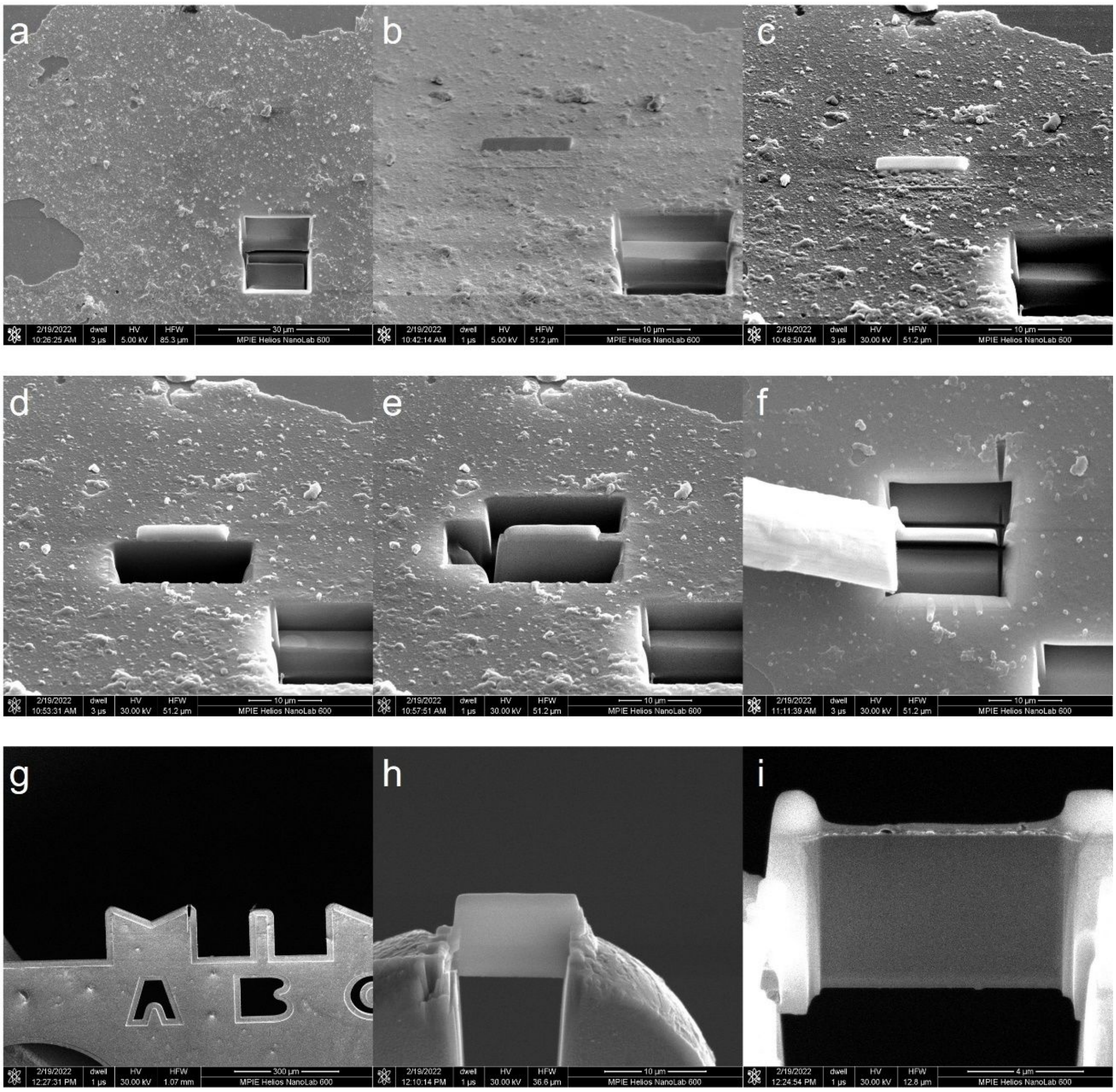

Fig. S10. TEM lamella preparation of the 10-cycled Si sample. (a) FIB/SEM surface image near the APT-site-lift-out region. (b) e-beam Pt/C deposition. (c) ion-beam Pt/C deposition. (d) & (e) Front-side and back-side cuts followed by the L-cut. (f) Attachment to a micro-manipulator. (g) FIB/SEM image of a commercial TEM Cu grid. (h) Mounted TEM lamella on the grid. (i) A final TEM lamella after the thinning process.

TEM measurement

TEM characterization of the cycled Si anode was performed in a JEM-2200FS TEM (JEOL) instrument operating at 200 kV. TEM images were acquired using a TemCam-XF416 pixelated scintillator-based complementary metal-oxide-semiconductor (CMOS) detector from Tietz Video and Image Processing Systems (TVIPS). We used Gatan Microscopy Suite® 3 Software to process TEM images, e.g. Fast-Fourier Transformation (FFT) of high-resolution TEM (HRTEM) images. The lamella specimen of the 10-cycled Si anode was titled close to Si [110] zone for the high resolution imaging of lattice fringes. Fig. S11a shows the interfacial regions between the Pt/C protection, the PECS-Cr, the electrolyte residuals, and the 10-cycled Si anode. Regions of interest have been highlighted in Fig. S11b to show local structure of the electrolyte residuals and the interface between the electrolyte residuals and the Si anodes. Fig. S12&13 present a variety of different defect structures observed in the 10-cycled Si anode.

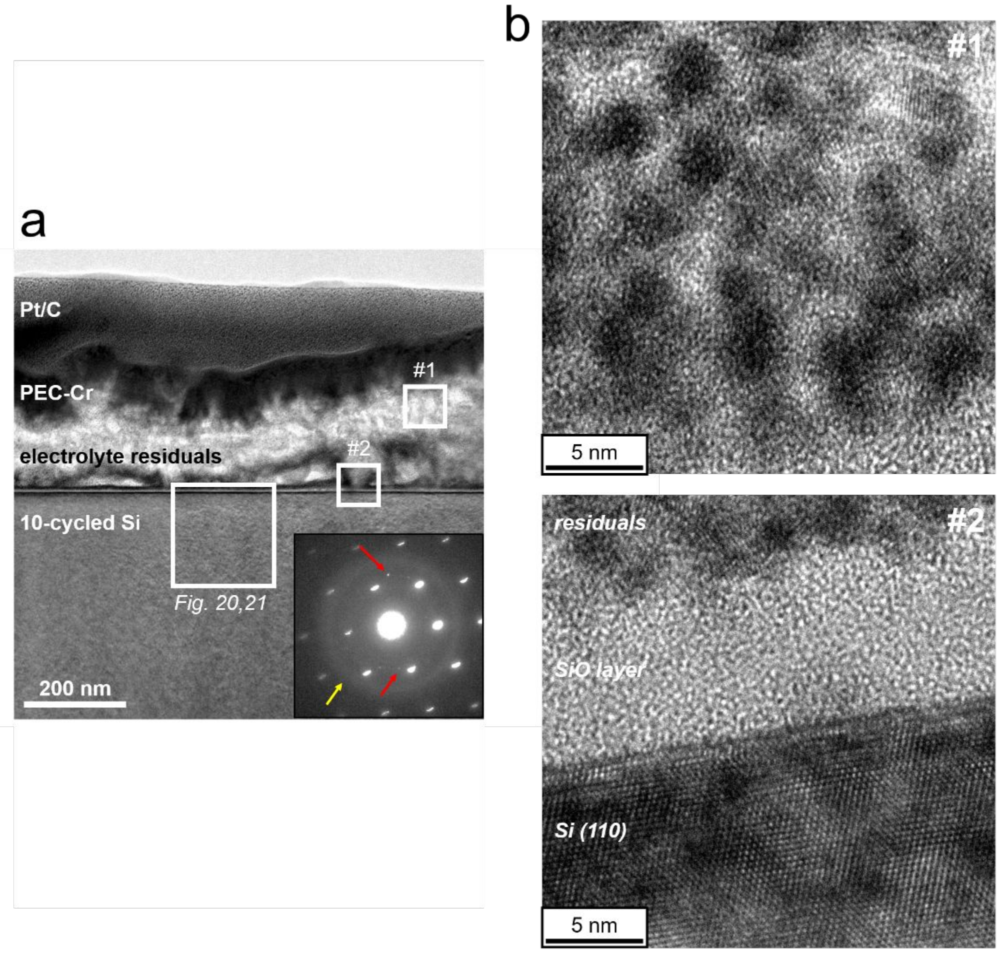

Fig. S11. TEM analysis of the 10-cycle Si. (a) Bright-field (BF) from the top-surface region and (b) high-resolution TEM (HRTEM) images of two small regions as marked with squares in (a). Inset image in **a** shows the complex diffraction patterns with amorphous ring (yellow) and un-identified diffraction points (red). The fast-Fourier transformation (FFT) patterns are measured along [110] Si zone axis.

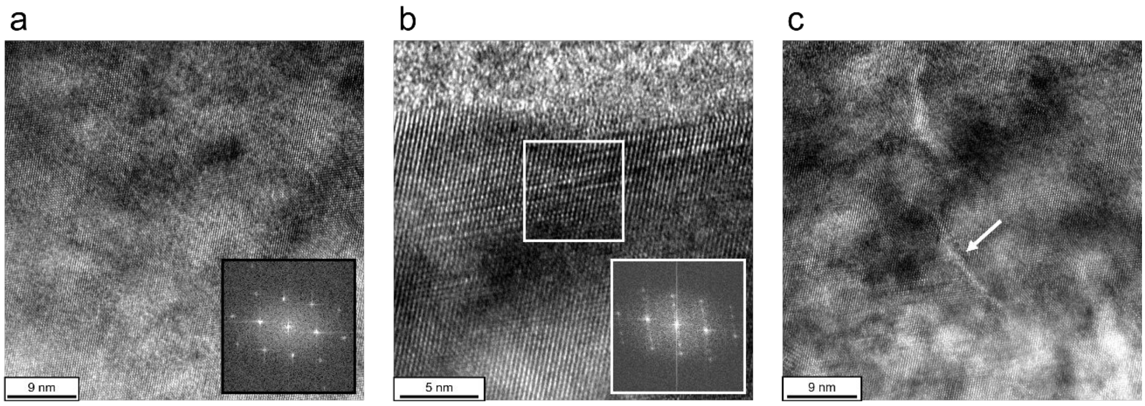

Fig. S12. HRTEM analysis of the 10-cycle Si. (a) Si region with a clear a single-phase Si[110] FFT pattern. (b) Defect sites at near-surface. (c) Defect sites within the Si anode.

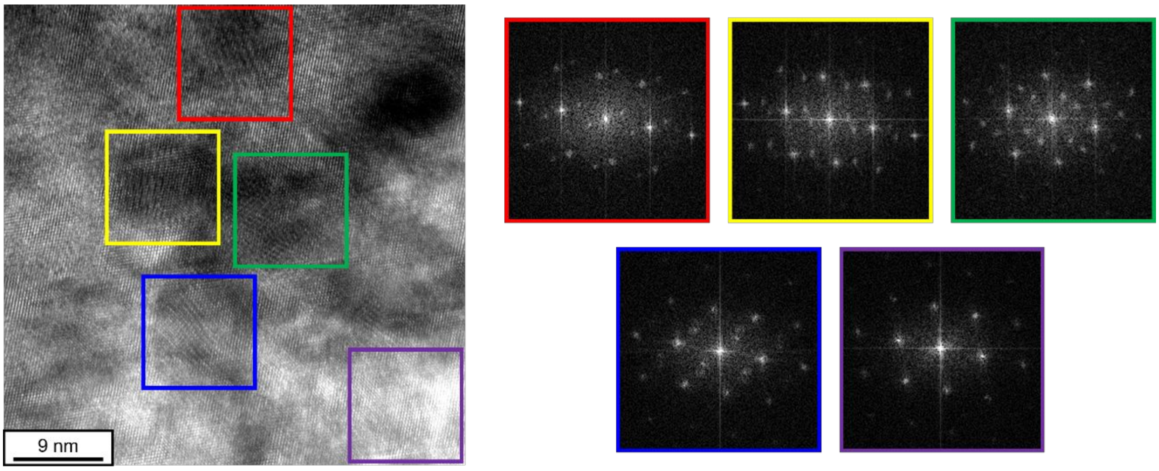

Fig. S13. HRTEM analysis of the 10-cycle Si. Note that these FFT patterns show different types of defects.

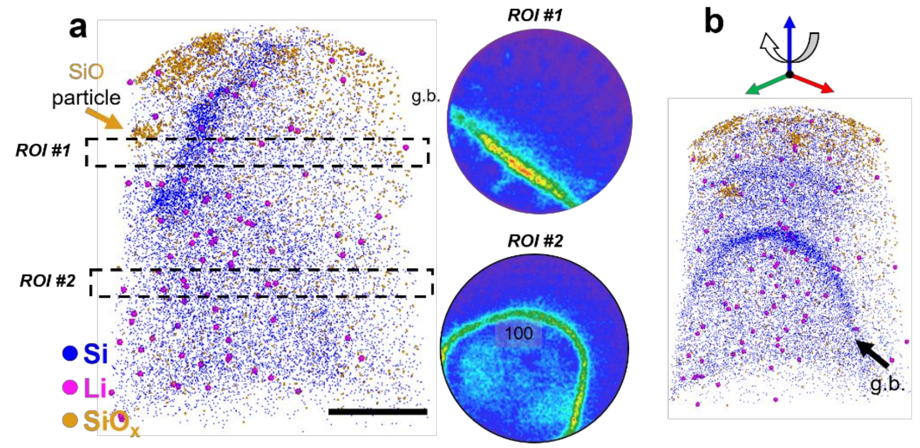

Fig. S14. (a) 3D atom map of the 25-cycle Si anode with Si ion density map. (b) 90°-rotated 3D atom map of 25-cycle Si anode (from Fig. 2f); a scale bar is 20 nm.

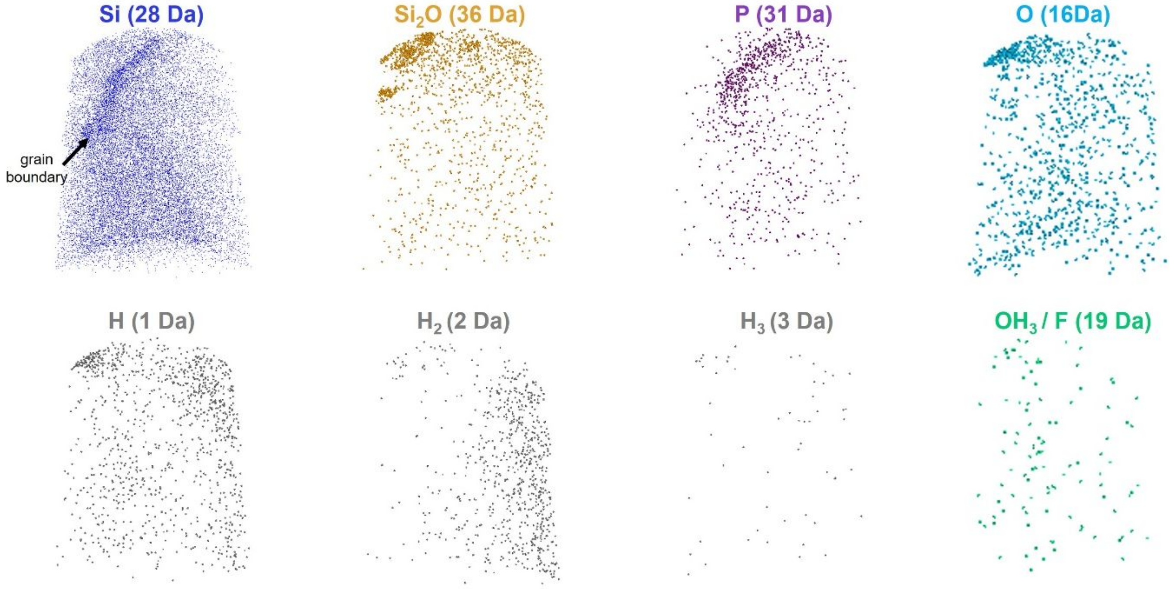

Fig. S15. 3D atomic distributions of Si, Si_2O, P, O, H, H_2, H_3, and OH_3/F species in the 25-cycled Si. It clearly shows that the peak at 31 Da is not related to H-Si peaks but different species (*i.e.* P). Note that O does not appear at region where P atoms locate. F^+ (19Da) overlaps with H_3O^+ and since there is no HF^+ and H_2F^+ peak in the mass spectrum, F may not be present in the Si.

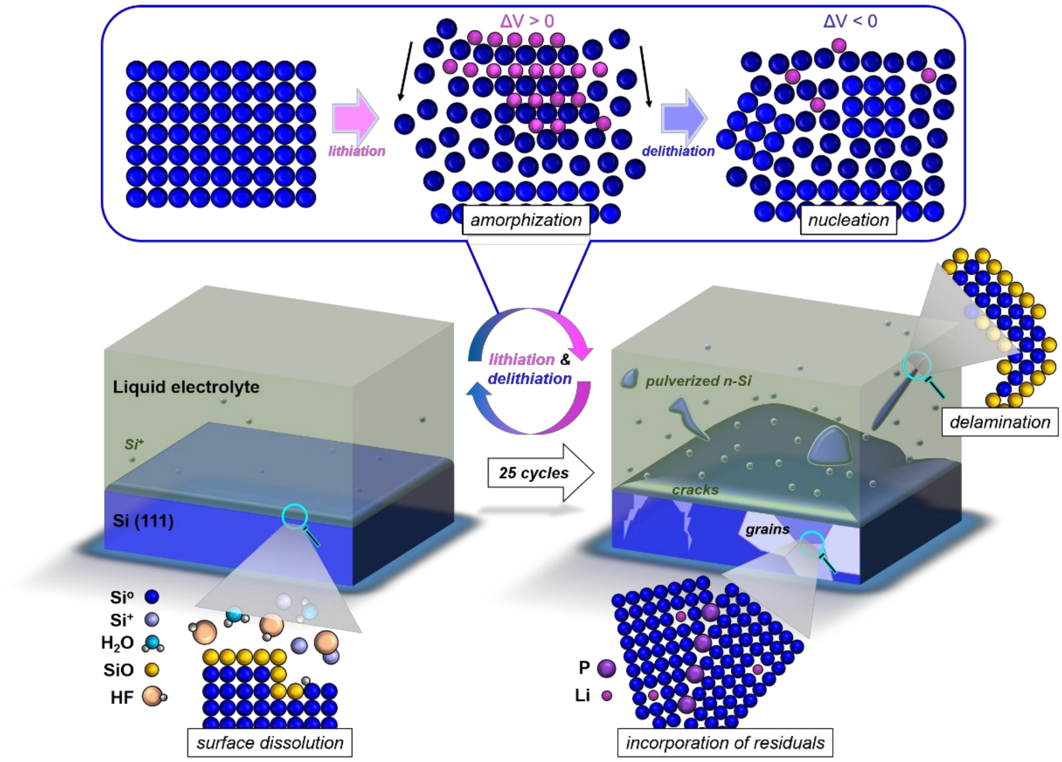

Fig. S16. Schematic illustration of Si anode failure: dissolution, delamination, implementation of electrolyte salt elements, and mechanical-stress relaxation.

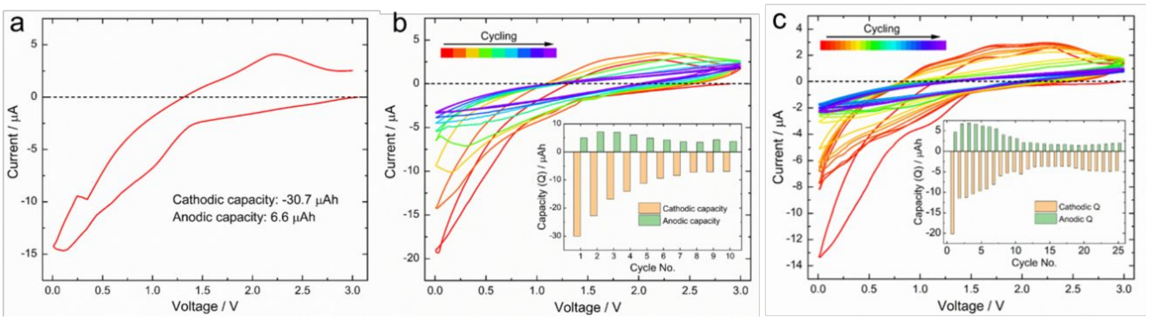

Fig. S17. The voltage profiles of the Li/Si cells after (a) 1, (b) 10, and (c) 25 cycles. Li/Si cells show similar electrochemical behavior during the CV scanning. From the insert figure in panel (b) and (c), evident cathodic capacity fading could be observed during the consecutive 10 and 25 cycles, respectively.

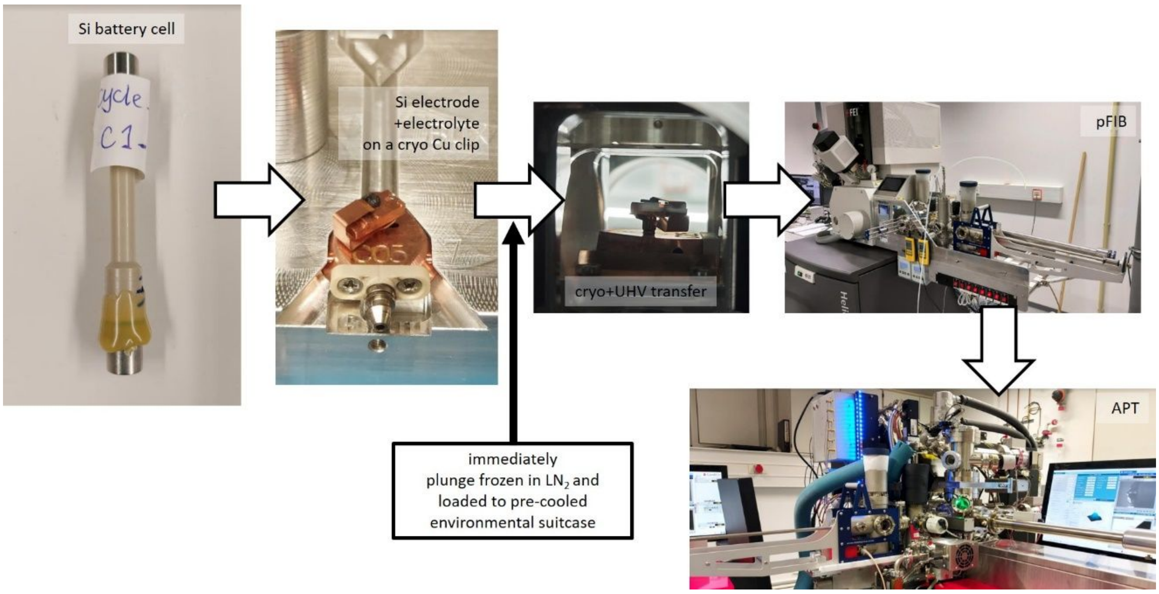

Fig. S18. The protocol of cryo-APT experiment. A battery cell was disassembled inside a N_2 glovebox and the interested cell was mounted on the Cu clip and immediately quenched into LN_2. Subsequently, the clip was loaded to the pre-cooled UHV carry suitcase (-190 ^oC and 10^{-9} mbar) and transferred to Gatan cryo-stage installed plasma-FIB. After final milling, the cold specimen was transferred back to the suitcase maintaining cryo-UHV conditions and was detached from the PFIB and mounted onto a LEAP 5000 XS atom probe system. Finally, the puck was transferred under cryo-UHV conditions to the atom probe analysis chamber. Details on the specific home-made installation are described in Ref.[8].

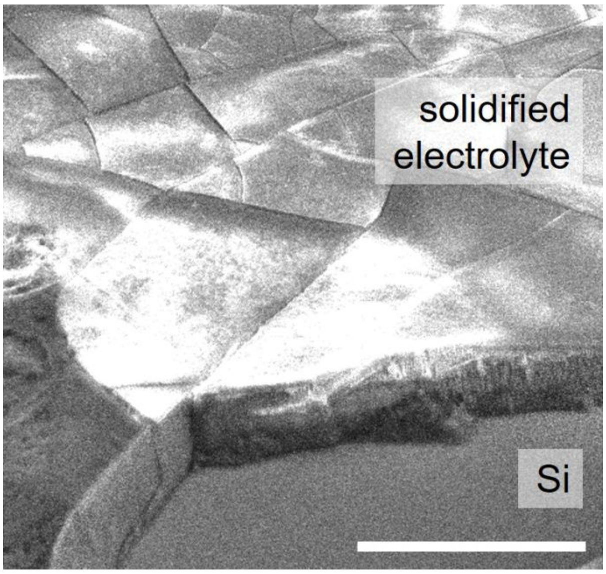

Fig. S19. Xe-plasma FIB/SEM image of frozen liquid electrolyte on the 1-cycle Si electrode. A white scale bar is 100 µm.

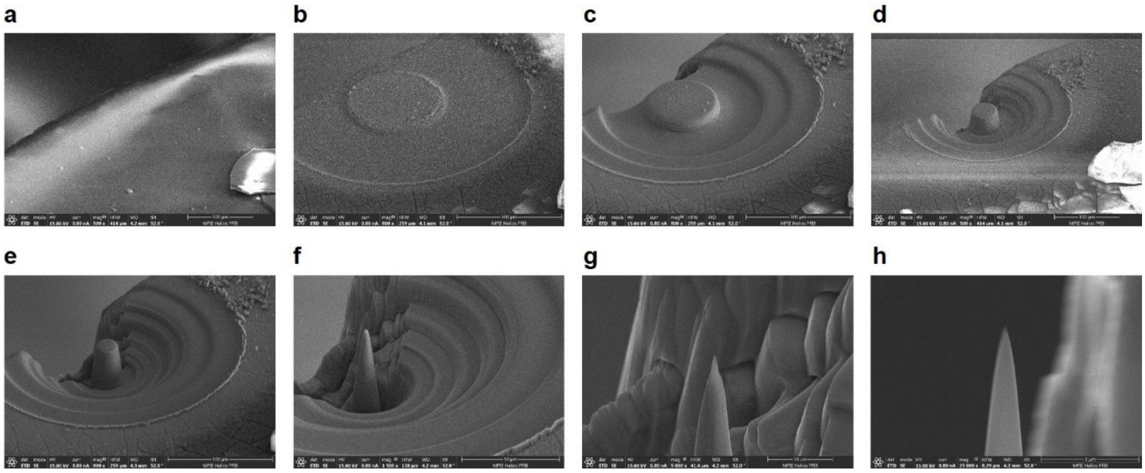

Fig. S20. APT specimen preparation of frozen liquid electrolyte/cryo-Si sample. The Halpin protocol was adapted to obtain a pillar shape[2]. (a) First, an ion-beam-circle pattern of outer diameter of 200 µm and inner diameter of 100 µm was set at 30 kV and 1.3 µA for 10 min. (b)-(e) Then patterns of outer and inner diameters were gradually reduced until inner diameters reach 30 µm with a depth of 50 µm. (f) The ion-beam current was set at 60 nA and the pillar was milled with a circle pattern (outer/inner = 50/10 µm) further down to fabricate into a toblerone shape. (g) Once a typical APT specimen geometry was obtained, the ion-beam current was reduced to 1 nA. (f) The final milling process was done at ion-beam current of 0.3 nA.

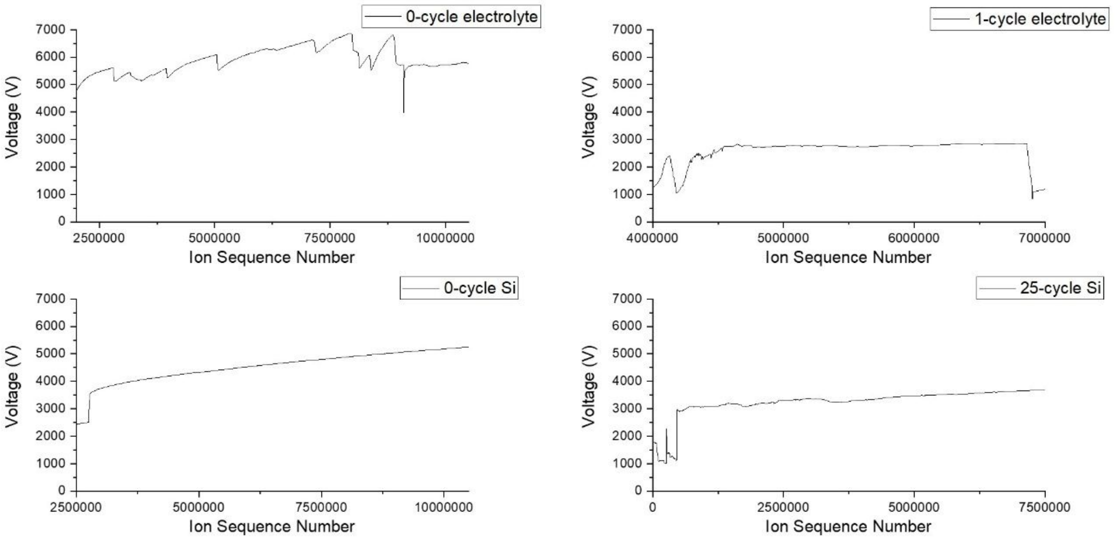

Fig. S21. Voltage curve of each cryo-measurement: 0-cycle electrolyte, 0-cycle Si anode, 1-cycle electrolyte, 1-cycle electrolyte containing n-Si debris, 25-cycle Si anode.

References

(1) Dong, K.; Osenberg, M.; Sun, F.; Markötter, H.; Jafta, C. J.; Hilger, A.; Arlt, T.; Banhart, J.; Manke, I. Non-Destructive Characterization of Lithium Deposition at the Li/Separator and Li/Carbon Matrix Interregion by Synchrotron X-Ray Tomography. *Nano Energy* **2019**, *62*, 11–19. https://doi.org/https://doi.org/10.1016/j.nanoen.2019.05.022.

(2) Halpin, J. E.; Webster, R. W. H.; Gardner, H.; Moody, M. P.; Bagot, P. A. J.; MacLaren, D. A. An In-Situ Approach for Preparing Atom Probe Tomography Specimens by Xenon Plasma-Focussed Ion Beam. *Ultramicroscopy* **2019**, *202*, 121–127. https://doi.org/https://doi.org/10.1016/j.ultramic.2019.04.005.

(3) El-Zoka, A. A.; Kim, S.-H.; Deville, S.; Newman, R. C.; Stephenson, L. T.; Gault, B. Enabling Near-Atomic–Scale Analysis of Frozen Water. *Sci. Adv.* **2020**, *6* (49), eabd6324. https://doi.org/10.1126/sciadv.abd6324.

(4) Hille, E.; Märk, T. D. Cross Section for Single and Double Ionization of Carbon Monoxide by Electron Impact from Threshold up to 180 EV. *J. Chem. Phys.* **1978**, *69* (10), 4600–4605. https://doi.org/10.1063/1.436411.

(5) London, A. J. Quantifying Uncertainty from Mass-Peak Overlaps in Atom Probe Microscopy. *Microsc. Microanal.* **2019**, *25* (2), 378–388. https://doi.org/DOI: 10.1017/S1431927618016276.

(6) Thompson, K.; Lawrence, D.; Larson, D. J.; Olson, J. D.; Kelly, T. F.; Gorman, B. In Situ Site-Specific Specimen Preparation for Atom Probe Tomography. *Ultramicroscopy* **2007**, *107* (2–3), 131–139. https://doi.org/10.1016/j.ultramic.2006.06.008.

(7) Giannuzzi, L. A.; Stevie, F. A. A Review of Focused Ion Beam Milling Techniques for TEM Specimen Preparation. *Micron* **1999**, *30* (3), 197–204. https://doi.org/https://doi.org/10.1016/S0968-4328(99)00005-0.

(8) Stephenson, L. T.; Szczepaniak, A.; Mouton, I.; Rusitzka, K. A. K.; Breen, A. J.; Tezins, U.; Sturm, A.; Vogel, D.; Chang, Y.; Kontis, P.; et al. The Laplace Project: An Integrated Suite for Correlative Atom Probe Tomography and Electron Microscopy under Cryogenic and UHV Conditions. *PLoS One* **2018**, *13* (12), e0209211. https://doi.org/10.1371/journal.pone.0209211.
